# A 3D Porous MXene/PNIPAAm Hydrogel Composite with Advanced Degradation Stability and Control of Electronic Properties in Air

**DOI:** 10.1002/advs.202516529

**Published:** 2025-11-14

**Authors:** Sitao Wang, Chen Jiao, Gerald Gerlach, Julia Körner

**Affiliations:** ^1^ Institute of Solid‐State Electronics Dresden University of Technology Helmholtzstraße 10 01069 Dresden Germany; ^2^ Leibniz Institute of Polymer Research Dresden Hohe Straße 6 01069 Dresden Germany; ^3^ Department of Electrical Engineering and Computer Science Leibniz University Hannover Schhneiderberg 32 30167 Hannover Germany

**Keywords:** degradation, electronic properties, gaseous environment, MXene, PNIPAAm hydrogel

## Abstract

This study reports the fundamental investigation of a composite material consisting of MXene (Ti_3_C_2_T_x_) and a stimulus‐responsive hydrogel (Poly(N‐isopropylacrylamide)–PNIPAAm). Thereby, the fabrication and comparison of pure MXene and composite samples featuring either a compact or a highly porous 3D microstructure, reveal unique properties with respect to: i) controllable 3D spatial arrangement of MXene instead of the prevalent stacked‐sheet structure, ii) reduction of oxidation‐induced degradation of MXene and substantially enhanced stability over the course of three months for the composite, and iii) tunable electronic states in response to gas interactions. Material characterization is conducted by scanning electron microscopy and rheology to assess the microstructural and mechanical properties, and in a chemiresistive measurement setup for the determination of electrical properties and the evaluation of the composite's potential for VOC sensing in a gaseous environment with the test analyte acetone. These investigations reveal material effects and properties that address some of the key MXene‐related challenges. Additionally, the interplay between the MXene and the hydrogel enables unprecedented opportunities for enhancing the sensing potential of stimulus‐responsive hydrogels, specifically in gaseous environments.

## Graphical abstract

This paper reports a fundamental study of the mutual benefits of the components in an MXene/PNIPAAm hydrogel composite with regard to enhanced degradation stability and tailorable electronic properties. The presented work focuses on the investigation of the fundamental material properties and ways for their modification. Furthermore, a chemiresistive sensing mechanism is explored for a future application of volatile organic compound detection.

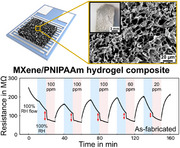

## 1 Introduction

MXenes are a class of 2D transition‐metal carbides/nitrides/carbonitrides that have gained substantial attention due to their versatility of composition and tuneability of properties, as well as many different potential applications, such as in energy storage and conversion, electronics, or biomedical contexts.^[^ ^1^, ^2^, ^3^ ^]^ However, despite extensive research efforts, fundamental challenges remain, which have been listed and ranked by experts in the field in 2021 as 32 major points to be addressed in the next decade.^[^ ^4^ ^]^ Among the top priorities are an improvement of chemical and temperature stability, the development of methods for creating fully 3D nanostructures with controlled alignment or orientation of MXene flakes, and the fundamental understanding and control of material properties such as electronic, optical, and magnetic.^[^ ^4^ ^]^

Reported approaches of addressing the degradation issue include defect passivation, chemical intercalation,^[^ ^5^, ^6^ ^]^ and, particularly for aqueous media, the combination with a polymer material.^[^ ^7^ ^]^ The latter can furthermore result in altered and enhanced material properties, which in turn are related to the exploration of new use cases. For example, the fabrication of MXene/polydopamine hybrid films with enhanced mechanical properties and ambient stability through the self‐polymerization of dopamine monomers on MXene surfaces has been reported.^[^ ^8^ ^]^ The uniform atomic‐level thickness of the polymer enables MXene to be structured in an idealized parallel manner, effectively preventing the penetration of oxygen and moisture. In another work, a conductive MXene/gelatin composite with excellent environmental stability and self‐adhesiveness for multifunctional sensor applications was demonstrated. The research findings suggested that gelatin molecules can be easily adsorbed onto the MXene sheets, forming a protective shield that prevents restacking and oxidation, resulting in a highly stable MXene/gelatin dispersion, as opposed to an aqueous MXene dispersion.^[^ ^9^ ^]^

In all of these cases, the polymer material is attached to or fabricated on the already formed MXene structure and assumes the role of a passivation layer. However, the combination of MXene and polymers can also be directed toward composite materials where the diverse properties of the MXene are employed to alter and engineer the polymer behavior, and, on the other hand, polymer functionalities benefit the MXene component.

In general, the creation of polymeric nanohybrids by incorporation of inorganic materials such as metal nanoparticles, metal oxides, graphene, or carbon‐based nanostructures^[^ ^10^, ^11^, ^12^, ^13^, ^14^, ^15^ ^]^ has been reported to lead to synergistic effects between the different phases in such a composite. The polymer matrix provides a high surface area with controllable and tunable pore size and distribution, as well as a scaffold for dispersion and arrangement of the additives, while the non‐polymeric components offer advantages such as electrical conductivity and versatile surface chemistry.^[^ ^16^, ^17^, ^18^ ^]^

It has already been demonstrated that the specific material composition and (surface) chemistry of MXenes and respective composites provide a promising basis for applications in the context of gas sensing and volatile organic compound (VOC) detection.^[^ ^19^, ^20^, ^21^, ^22^ ^]^ For this purpose, the material needs to be specifically tailored to reach high sensitivities, for example, by partial oxidation and spatial arrangement of the MXene sheets.^[^ ^23^, ^24^, ^25^ ^]^

Another material candidate for sensing and actuation applications, in particular in biomedical contexts, is stimulus‐responsive (smart) hydrogels.^[^ ^26^, ^27^ ^]^ This is a specific class of polymers whose key feature is a reversible volume change as a reaction to external stimuli such as light,^[^ ^28^, ^29^ ^]^ pH,^[^ ^30^ ^]^ temperature,^[^ ^31^ ^]^ ionic strength,^[^ ^32^ ^]^ and biological molecules.^[^ ^33^ ^]^

However, the volume change of smart hydrogels is mainly mediated by the absorption and release of liquid, which has largely limited the application scope to aqueous environments and humidity sensing.^[^ ^34^, ^35^ ^]^ Only recently has it been explored how this boundary condition can be overcome to enable harnessing of the selective and sensitive volumetric response for volatile organic compound (VOC) detection in gaseous environments.^[^ ^36^, ^37^, ^38^, ^39^ ^]^ In prior work, we have successfully demonstrated a novel approach to specifically tailor the structure of polymer networks for long‐term stable porosity in air with largely varying humidity (5% to 100%), and the applicability for the detection of the organic test analytes acetone and isopropanol. The specific smart hydrogel suitable for the chosen test gases was found to be Poly(N‐isopropylacrylamide) (PNIPAAm), but the developed porosity engineering techniques are of a general nature and applicable to polymer materials in general.^[^ ^38^, ^39^ ^]^

Please note that PNIPAAm is, in principle, a temperature‐responsive hydrogel that undergoes a volume‐phase transition in liquid environments at a lower critical solution temperature (LCST) of 33 °C.^[^ ^40^, ^41^ ^]^ However, this responsiveness is not utilized for the use of VOC detection in air, where a change of the hydrophilic‐hydrophobic balance in the polymer network is only expected to have a minimal effect due to the absence of a solution environment. Instead, the functional groups of the polymer directly interact with the VOC, and a detailed explanation of this mechanism is presented in prior work.^[^ ^38^ ^]^ Furthermore, all investigations reported in the following have been conducted under cleanroom conditions at a controlled temperature of (20–22) °C, which is significantly below the LCST of PNIPAAm hydrogel.

While the potential of PNIPAAm for gas sensing has been demonstrated, the aforementioned mechanism of direct interaction between the polymer's functional groups and the VOC poses challenges with regard to i) limited sensitivity and swelling response due to the adsorption and desorption dynamics of the organic compounds with the polymer, and ii) the reliable transduction of the smart hydrogel's volume change response.

In both aspects, MXene is an ideal material to be combined with smart hydrogels to i) enhance the responsiveness for VOCs, and ii) equip the intrinsically insulating polymer with electrical conductivity to extend the array of potential transduction concepts in the sensing context. To date, the majority of reported work on polymer‐MXene composites has focused on either one of the components as the active material, while the other one is used as a support, e.g., scaffold, encapsulation, or conductivity enhancer.^[^ ^42^ ^]^ Furthermore, the combination of MXene with hydrogels has been studied comparatively little, as evidenced by recent reviews.^[^ ^43^, ^44^, ^45^ ^]^ An overview is provided in **Table** **1** and ref. [45]. For PNIPAAm hydrogel, no combination with MXene for gas sensing, in particular where the responsiveness of both materials is considered, has been reported to date.

**Table 1:** Overview of reported studies on MXene‐polymer composites and their applications.

Material	Application	Refs.
MXene on 3D polymer framework	VOC gas sensing Sensing material: MXene; polymer (PVA/PEI) as 3D scaffold	[46]
MXene/polyurethane core‐sheath fibers	VOC gas sensing MXene: VOC sensing + conductivity; polyurethane: stretchability	[47]
MXene/PNIPAAm composite	Soft manipulators and strain sensors Hydrogel: softness; MXene: conductivity + photothermal response enhancer for hydrogel actuation	[48]
Cyclodextrin‐encapsulated MXene/PNIPAAm composite	Absorbent for phenols in waste water Both combined: enhanced binding of target; cyclodextrin as 3^rd^ component to extent material lifetime	[41]
MXene/PNIPAAm composite	Smart compression sensor (temperature + stress) Hydrogel: softness + stretchability; MXene: conductivity	[49]
MXene nanocomposite polymers	Conductive 3D printable polymer for soft electronics MXene: conductivity; polymer: printable resin	[50]
MXene/PNIPAM composite	Strain + temperature sensor MXene with polydopamine (PDA) coating: conductivity; PNIPAM: temperature sensitivity	[51]
MXene/PAAm composite	Detection of liquid and gaseous hexanal MXene: conductivity; hydrogel: solubility matrix for target analyte	[52]

In the work presented here, we have created a novel composite material comprising the smart hydrogel Poly(N‐isopropylacrylamide) (PNIPAAm) and MXene (Ti_3_C_2_T_x_) that, for the first time, enables the exploration of the phenomenon of a mutual beneficial interaction and combination of properties toward new functionalities, with an exemplary application in gas sensing. It relies on a recently developed process of structural porosity engineering of polymers^[^ ^38^, ^39^ ^]^ to achieve an interplay between both components.

Thereby, the MXene, on the one hand, alters the mechanical and electrical properties of the hydrogel, also with respect to VOC sensing mechanisms. On the other hand, the interplay with the polymer enables the necessary MXene modifications beneficial for gas sensing (partial oxidation, 3D spatial arrangement) mentioned above and, furthermore, results in unprecedented opportunities for addressing some of the challenges of MXenes mentioned in the beginning. The unique features of the composite material include i) the spatial arrangement of MXene flakes in a fully 3D structure instead of the usual stacked‐sheet configuration, ii) control over the electronic response to a gas interaction on the range from metallic to semiconducting by tailoring the composite's porosity, and iii) a reduction of oxidation‐induced degradation of MXene and unprecedented stability over the course of three months.

While integrating MXene with hydrogel materials is not a novel concept, the presented dried composite sample stands out by offering a tunable porous structure for gas sensing that maintains its stability in any kind of humid VOC environment, a feature that has not been documented in the current literature. Additionally, the fabrication process is significantly simpler and more efficient compared to other studies on porous MXene composites.

In the following, the fabrication and characterization of pure MXene and MXene/PNIPAAm composite samples are described with respect to the fundamental material properties and their potential for VOC sensing applications. Besides scanning electron microscopy and rheology for assessing microstructure and mechanical properties, chemiresistive measurements are employed to gain insights into the electronic behavior of the samples in response to exposure to acetone as an exemplary test VOC. Chemiresistive sensing utilizes the change of electrical impedance of an electrode structure (usually interdigitated electrodes – IDEs) containing the sensing material as a dielectric medium. The electrical properties of the sample material are affected by the interaction with an analyte (or other stimulus in the case of a hydrogel), which in turn is detected and monitored by the IDE's impedance.

The analysis of the response to the exemplary organic compound acetone enables the assessment of the electronic states as well as the degradation stability with regard to oxidation of the MXene phase of the MXene/PNIPAAm composite. The comparison with pure but 3D arranged MXene furthermore reveals the role of the hydrogel and the interplay between the two components, indicating a novel path toward adjustable and controlled electronic properties and a tunable spatial MXene arrangement.

These results highlight the potential of combining MXene and PNIPAAm hydrogel for mutually beneficial material properties and build the foundation for a prospective application in VOC sensing with chemiresistive readout.

## 2 Results and Discussion

### 2.1 Overview of Studied Materials

To assess the performance of individual components, pure MXene (Ti_3_C_2_T_x_) and MXene/PNIPAAm composite materials were synthesized as bulk (1 cm × 0.5 cm × 0.2 cm) and as thin film samples on interdigitated electrodes (IDEs). Bulk samples were fabricated with molds (glass slides of 76 mm × 52 mm with a 500 µm thick Teflon spacer), and the samples were cut into smaller pieces of ≈0.001 mg and ≈0.004 mg for pure MXene and the composite, respectively, for analysis. The thin films were created by drop‐casting of 4 µL of precursor solution (pure MXene) or by molding (composite samples) on the desired target substrate (Figure S1, Supporting Information). The IDEs in the study comprise either a gold or platinum metallization layer (same electrode pattern) and different base materials (polyimide, glass, ceramics) to match the material properties of the attached material (either pure MXene or the composite), ensure good adhesion, and therefore a reliable electrical impedance read‐out.

In all cases where MXene was used, a corresponding suspension of a concentration of 30 mg mL^−1^ was first made from powder and then used for further processing. This concentration was chosen based on prior experiments, which showed that 30 mg mL^−1^ is the highest MXene content that still enables a successful polymerization in combination with the target hydrogel PNIPAAm. Higher MXene contents cause incomplete polymerization and subsequent disintegration of the samples. Lower MXene concentrations are studied in the context of the mechanical properties of the composite as described in Section 2.3.5.

To induce porosity in the composite material, poly(ethylene‐glycol) (PEG) of molecular weight 10000 (FLUKA, Germany, used as received) was employed, based on the results of a previous study.^[^ ^39^ ^]^

Samples were characterized with respect to their appearance, microstructure, and mechanical properties by optical and scanning electron microscopy, as well as rheology. EDX analysis was performed to determine sample composition. To study the chemiresistive performance of pure MXene and composite samples, IDEs equipped with the respective material were placed in a sealed chamber where desired environmental conditions could be created (Figure S2, Supporting Information), and the IDE impedance was measured with a digital multimeter (Fluke 45 Dual Display Multimeter, Germany). In the presented studies, acetone is used as the exemplary test analyte to enable a comparison with prior work.^[^ ^38^, ^39^ ^]^ Furthermore, this setup is also employed to assess the degradation stability of the investigated samples.

**Table** **2** provides an overview of the studied sample types and used characterization methods, since not all techniques have been applied to every sample type due to feasibility. All investigations have been performed for at least three samples of each type to ensure reproducibility and reliability of the reported findings. Please refer to the Supporting Information for details about synthesis and characterization.

**Table 2:** Overview of studied samples and applied characterization technique for different sample types. Used abbreviations: FD ‐ freeze‐dried; AD ‐ air‐dried; PEG ‐ poly(ethylene‐glycol); OM ‐ optical microscopy; SEM ‐ scanning electron microscopy; EDX ‐ energy‐dispersive X‐ray spectroscopy; Rheo ‐ rheology; CR ‐ chemiresistive (short‐term, i.e., as‐fabricated and long‐term analysis with repeated testing over weeks).

Category	Specific properties	OM	SEM	EDX	Rheo	CR short	CR long
**Pure Mxene**	Bulk, FD	✓	✓	✓			
	On‐chip, AD	✓	✓	✓		✓	
	On‐chip, FD	✓	✓	✓		✓	✓
**MXene/PNIPAAm composite**	Bulk, FD, with PEG	✓	✓	✓	✓		
	Bulk, FD, without PEG	✓	✓	✓			
	On‐chip, AD, with PEG	✓	✓	✓		✓	
	On‐chip, FD, with PEG	✓	✓	✓		✓	✓

### 2.2 Analysis of Pure MXene

**Figure 1:**
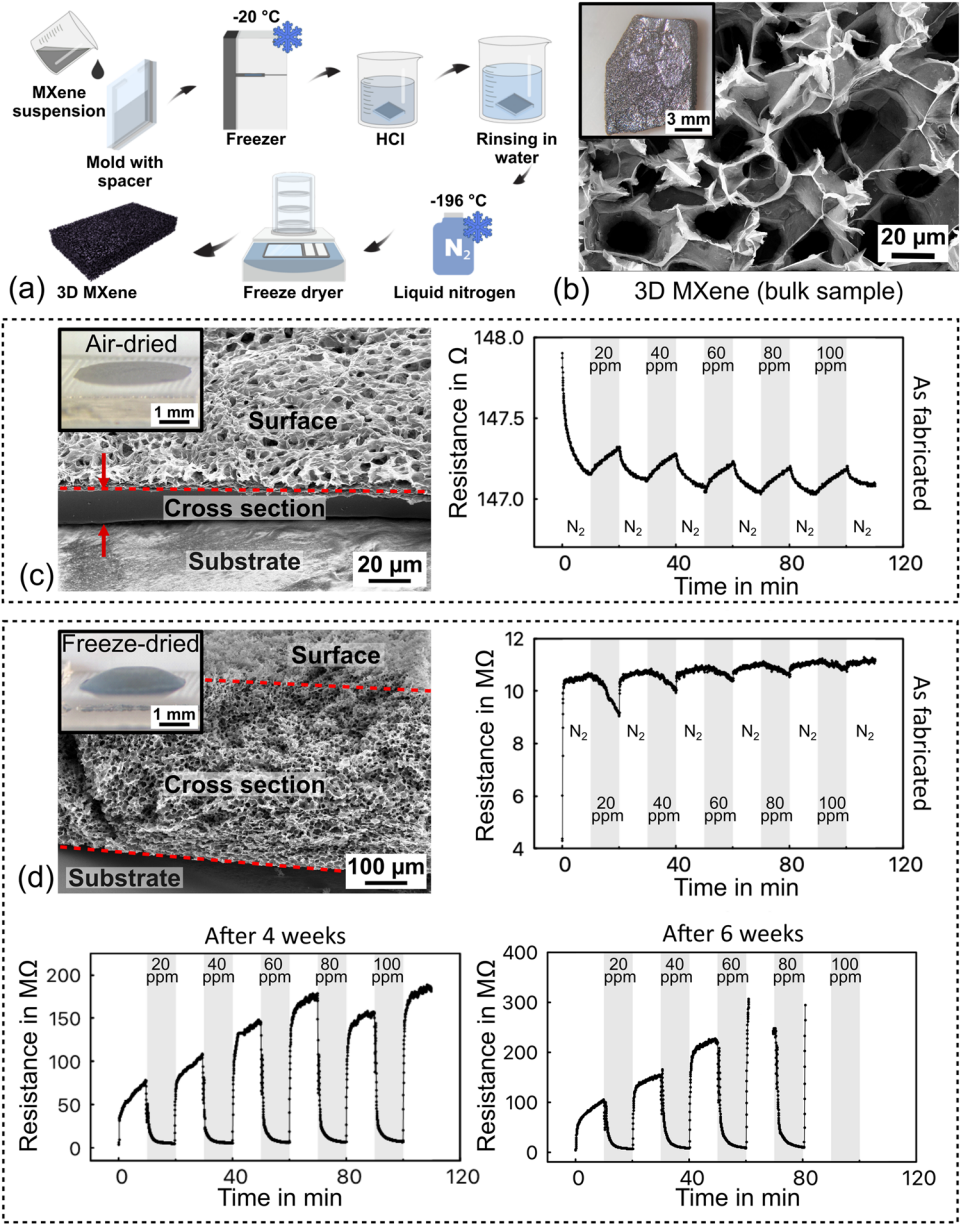
3D bulk pure MXene: a) fabrication process, b) optical microscope (inset), and SEM images of the obtained material. c, d) Optical microscope (insets) and SEM images of samples on IDEs (fabricated by drop casting) and the respective results of the chemiresistive measurements in (20–100) ppm gaseous acetone with a dry nitrogen background: c) air‐dried compact MXene and d) freeze‐dried 3D porous MXene. The 3D sample was repeatedly measured over the course of six weeks. IDE measurements were performed on as‐fabricated samples without prolonged storage. Both samples were fabricated using the same volume of MXene suspension and a concentration of 30 mg mL^−1^.

In the following, the results of the pure MXene samples (bulk and on IDE) are described to establish a baseline for comparison with the composite. **Figure** **1** depicts the fabrication process, SEM images, and IDE output signals for the respective samples.

Please note that the fabrication steps in Figure 1a are shown for the bulk sample, but were carried out in a similar way for the fabrication on IDE. In this case, instead of applying the MXene suspension to a mold, it was drop‐casted on the IDE.

#### 2.2.1 Microstructure

It needs to be noted here that the freezing temperature during the ice‐templating process (‐20 °C, exemplarily indicated in Figure 1a) can significantly influence the appearance and mechanical stability of the resulting MXene structure (Figure S5, Supporting Information). Samples created by freezing in liquid nitrogen (‐196 °C) resulted in a fragile scaffold, with the material falling apart. In addition, during quenching, the large temperature difference led to rapid nucleation and directional growth of ice crystals, leaving behind channel‐like voids across the cross‐section, with an orientation depending on the applied temperature gradient.^[^ ^53^ ^]^ In contrast, when freezing at a higher temperature (‐20 °C), the slow cooling rate allows ice crystals to develop omnidirectionally, leaving uniform cellular structures after drying.^[^ ^54^ ^]^ Furthermore, this also promotes the formation of integral sections within the highly porous network, which are stiffer compared to the heterogeneous structures containing one‐directional channel‐like pores achieved at ‐196 °C. Therefore, a freezing temperature of ‐20 °C has been selected in this study for sensing applications. In SEM, the bulk pure MXene structure frozen at ‐20 °C exhibits a honeycomb‐like porous network with a dark black color and shiny metallic features on the surface, as shown in Figure 1b. The appearance under the optical microscope shows a uniformly gray and shiny coloring.

Figure 1c,d depict the pure MXene on IDEs that have been treated by two different procedures after fabrication and rinsing. In one case (Figure 1c), the sample was left to air‐dry under cleanroom conditions (22 °C, 45% RH), while in the second case, freeze‐drying at ‐196 °C was applied (Figure 1d). This comparison was done to clarify if the pure MXene responds to different drying procedures in the same way as the PNIPAAm hydrogel (see ref. [39].

In accordance with the previous investigations of hydrogel, air‐drying of the MXene results in an irreversible collapse of the porous network. Despite having a rugged surface morphology with recognizable pores, the capillary forces generated during water evaporation lead to the stacking of material layers with a reduced thickness of several microns and a densely packed internal structure.^[^ ^55^ ^]^ In contrast, the 3D MXene network is preserved through freeze‐drying at ‐196 °C in the same way that has been established for hydrogel material in previous studies.^[^ ^39^ ^]^ The resulting MXene layer thickness amounts to several 100 µm, with the same amount of solution being used as in the air‐dried case. The SEM image shows a homogeneous pore size and distribution with honeycomb‐shaped voids along the cross‐section (Figure 1d). The average pore diameter is 86 µm and has been obtained through analysis of SEM images with *ImageJ* (refer to the Supporting Information for details).

#### 2.2.2 IDE Impedance Measurements

Chemiresistive measurements were carried out in an acetone atmosphere with concentrations ranging from 20 ppm to 100 ppm (by adding a corresponding amount of liquid solvent to the chamber as described above) and using dry nitrogen as the background and purge gas. The results for air‐ and freeze‐dried IDE samples are also shown in Figure 1c,d. The dense MXene layer obtained by air‐drying exhibits a resistance increase when exposed to an acetone environment, consistent with findings from other published work.^[^ ^56^, ^57^ ^]^ However, the values show almost no dependence on the acetone concentration. The porous 3D MXene sample exhibits a reversed response, i.e., a resistance decrease, and a dependence on the acetone concentration. The resistance drop became more pronounced in the second test that was conducted four weeks after the initial test and subsequent storage under ambient cleanroom conditions, demonstrating a clearer correlation: with increasing acetone concentration, a larger resistance decrease occurred. However, the sample became immeasurable in the third test after six weeks of storage, as the resistance value increased to a very high level.

**Figure 2:**
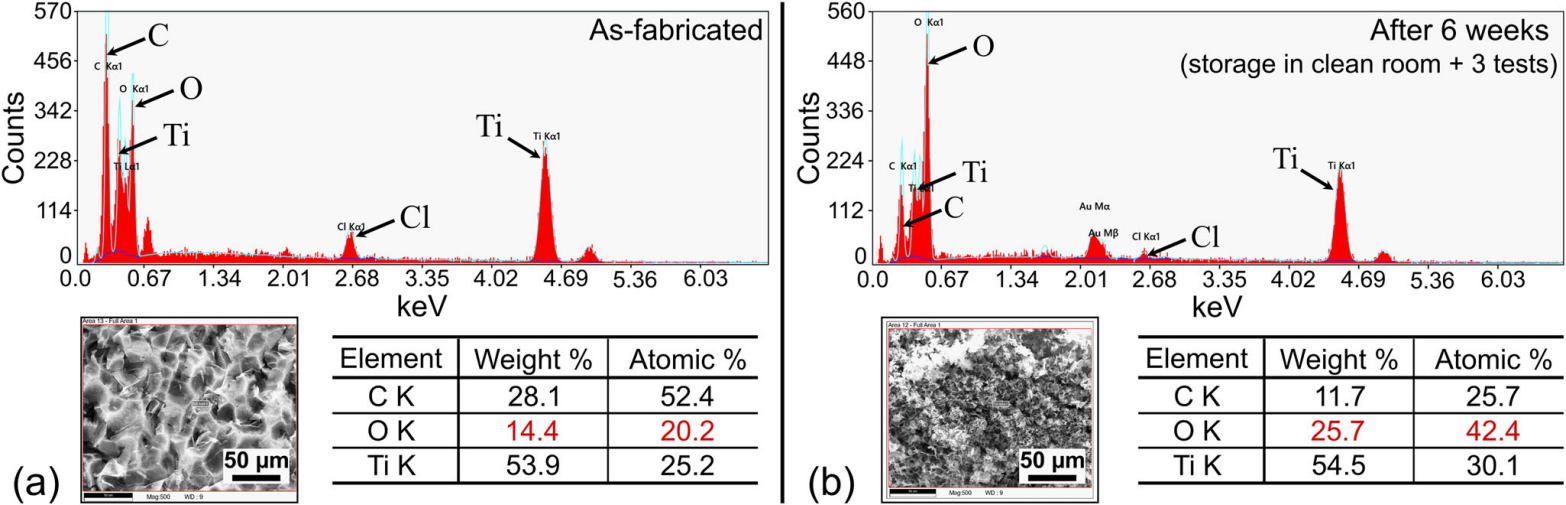
EDX analysis results of 3D (freeze‐dried) MXene samples: a) as‐fabricated, b) after three tests in acetone atmosphere over a period of six weeks. In between impedance measurements, the sample was stored under cleanroom conditions. Please note that the SEM images in both subfigures have been taken from the same sample but at a different location. Morphological differences are attributed to normal fabrication‐induced variations.

EDX analysis was performed on the 3D (freeze‐dried) pure MXene sample right after fabrication and after exposure to an acetone atmosphere for three tests to discern any differences. As depicted in **Figure** **2**, the oxygen content of the as‐fabricated MXene was notably lower than that of the sample after tests in acetone, indicating oxidation of the material. Please note that the associated SEM images might create the impression of morphological changes within the same sample in the as‐fabricated state and after repeated testing. However, analysis of various locations on the same and different samples indicates that varied appearances are likely due to normal fabrication‐induced variations. No consistent or reproducible alteration was observed.

### 2.3 Analysis of MXene/PNIPAAm Composite

**Figure 3:**
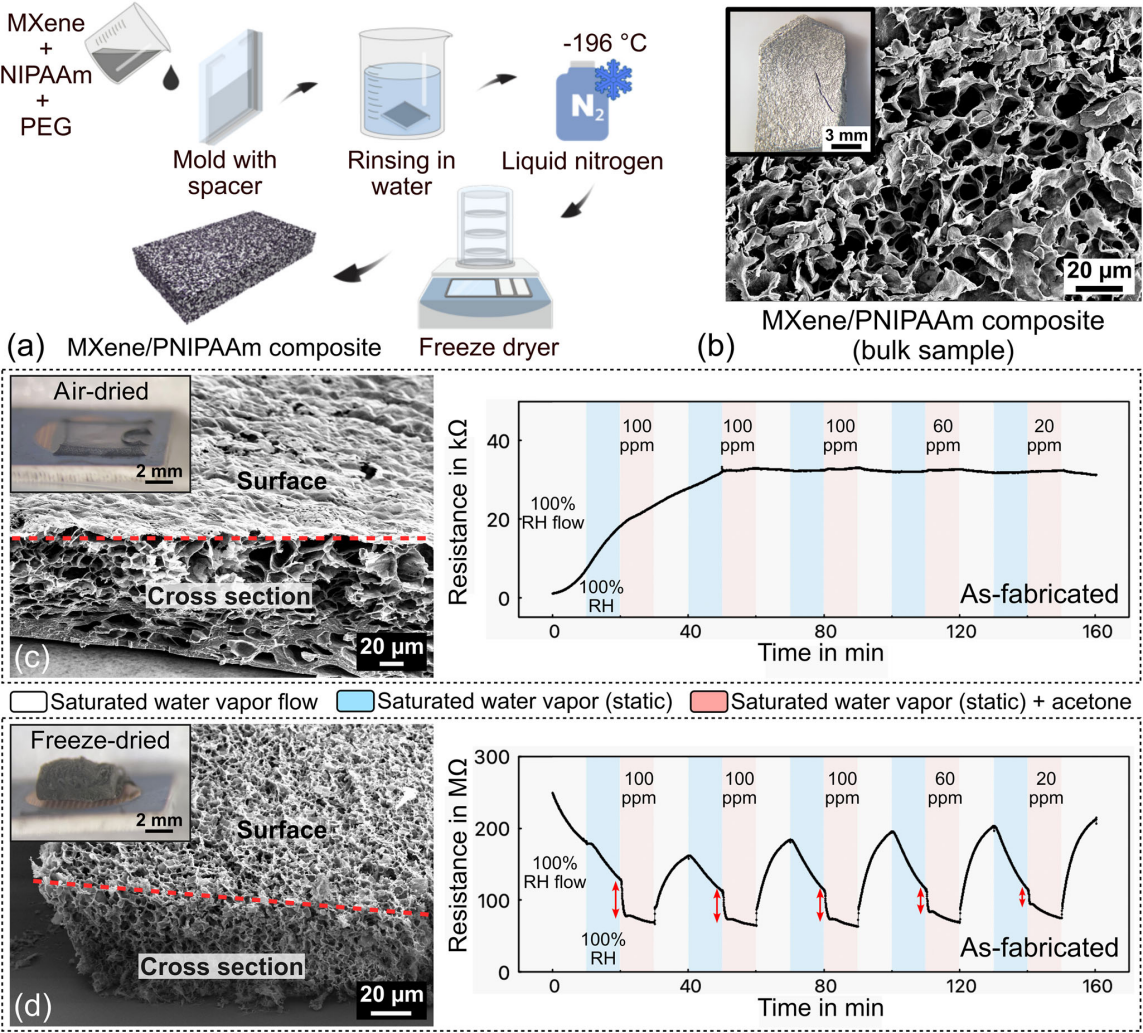
Porous MXene/PNIPAAm composite: a) Fabrication process, b) bulk sample images (inset: optical microscope; main: SEM). Due to the synthesis process, the MXene is fully integrated with the polymer matrix, and consequently, no individual MXene sheets can be identified. The homogeneous MXene distribution is instead evidenced by the EDX‐mapping of the titanium element depicted in Figure 4. Optical microscope (inset), SEM images, and chemiresistive analysis of MXene/PNIPAAm composite samples on IDE (fabricated by molding): c) air‐dried compact and d) freeze‐dried porous composite in response to gaseous acetone with a high humidity background. IDE measurements were performed on as‐fabricated samples without prolonged storage. Both samples were fabricated with the same amount of precursor solution and a MXene concentration of 30 mg/mL.

The MXene/PNIPAAm composite with integrated PEG was fabricated as depicted in **Figure** **3**a. The porogen PEG is necessary to create a porous hydrogel structure and, in contrast to ice crystal formation, it enables a more controlled approach as found in previous studies.^[^ ^35^ ^]^ In contrast to the pure MXene, no freezing and HCl treatment are required, as the polymer provides the scaffold and linkage for the MXene flakes.

#### 2.3.1 Microstructure

In contrast to the almost completely grayish/black color of the pure MXene sample (Figure 1b), the MXene/PNIPAAm composite shows a black and white appearance under the optical microscope, as depicted in Figure 3b, indicating the presence of the polymer within the network. Furthermore, instead of the semi‐transparent thin walls of pure MXene structures, the composite's morphology is more akin to the plain polymer, with embedded MXene flakes integrated into the polymeric matrix.^[^ ^39^ ^]^ For reference, optical microscope and SEM images of pure PNIPAAm hydrogel featuring a clear white color are depicted in Figure S6 (Supporting Information).

The determination of the composite's pore size is challenging due to the formation of an overlapping, irregular network of the polymer walls with the embedded MXene nanosheets. This effect is attributed to the relatively high concentration of the used MXene suspension. Overall, the pore diameter in the composite is ≈(3‐4) µm as obtained from the analysis of SEM images with *ImageJ* (refer to the Figure S4, Supporting Information for details). However, as demonstrated in prior work,^[^ ^39^ ^]^ it can be tailored by the molecular weight and concentration of the used porogen (PEG 10000 in the presented case) as well as the conditions during synthesis and freeze drying. Since the focus of the current work is on the fundamental analysis of the novel composite's properties, a comprehensive investigation of the controlled porosity is beyond the scope.

The bulk sample has been freeze‐dried for microstructural analysis. For the on‐chip samples, two different drying methods (air‐ and freeze‐drying) have been employed to study the influence on the sample morphology and consequently the gas sensing performance. By comparing the SEM images in Figure 3c,d, it is clearly evident that freeze‐drying results in a finer porous structure, while air‐drying leads to larger pores that are partially deformed and collapsed due to the slow evaporation of water molecules during drying. The most notable difference occurs on the sample surface: the freeze‐dried material is equally porous on the surface and on the inside with interconnected pores throughout its entirety. In contrast, the surface of the air‐dried sample forms a more skin‐like closed layer that separates the inner porous network from the surroundings. These findings are consistent with previous studies of PNIPAAm.^[^ ^39^ ^]^ Further detailed SEM images, as well as a comparison to the pure MXene samples, can be found in the Supporting Information (Figure S7, Supporting Information).

With regard to the overall sample appearance (inset optical microscope images in Figure 3c,d), air‐drying results in a very thin layer on the IDE, with some material accumulating around the edges. This accumulation can be attributed to the coffee ring effect that has been observed for the drying of polymer droplets containing solid‐phase colloidal particles.^[^ ^58^ ^]^ In contrast, freeze‐drying preserves the structure and thickness of the original water‐containing hydrogel. Please note that the same amount of precursor solution was used for both samples, resulting in a comparable watery sample thickness directly after synthesis.

#### 2.3.2 EDX Analysis

**Figure 4:**
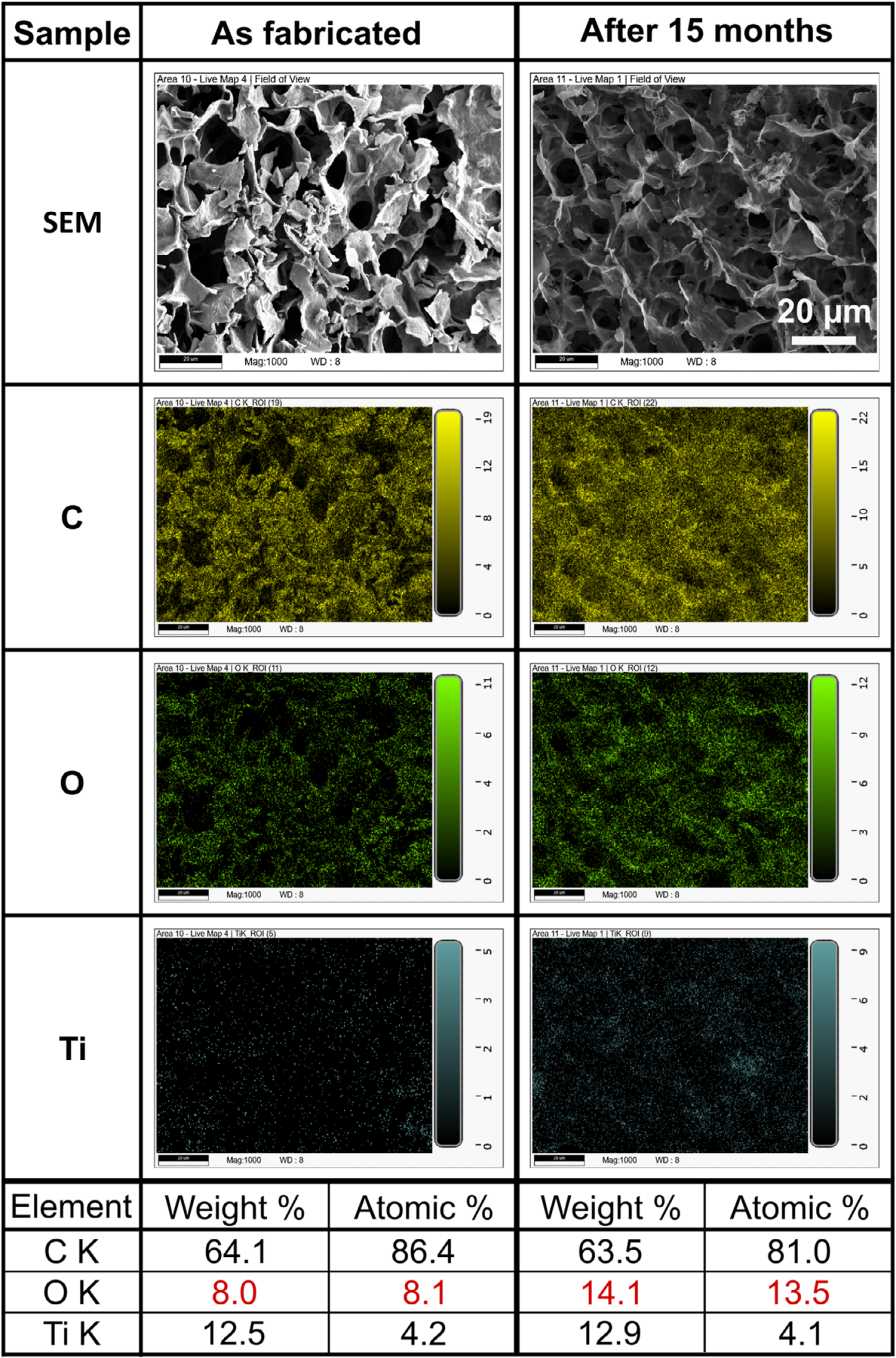
Energy‐dispersive X‐ray spectroscopy (EDX) mapping results of an as‐fabricated bulk MXene/PNIPAAm composite sample (left column), and an on‐chip composite sample after 15 months of repeated testing in acetone with high humidity and storage under ambient cleanroom conditions in between (right). The scale bar applies to all images. Extracted weight percentage and atomic percentage values for the relevant elements carbon (C), oxygen (O), and titanium (Ti) are summarized in the table.

**Figure 5:**
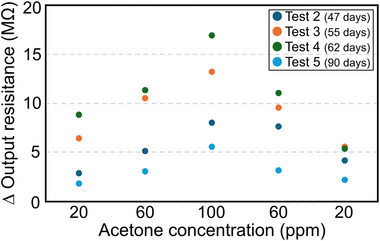
Acetone response of repeated testing of the same MXene/PNIPAAm composite sample on IDE for five separate tests (details in Tables S2 and S3, Supporting Information). The numbers in brackets denote the time after sample fabrication, i.e., the “sample age”. The output resistance change is calculated as the step height of the initial sharp drop, as indicated by arrows in Figure 3d. Please note that the first test has been excluded as it is considered a conditioning of the sample.

Since SEM images provide only surface‐morphological information with embedded MXene sheets remaining mostly invisible, EDX was used to confirm the distribution of MXene within the polymer matrix. The results depicted in **Figure** **4** verify the homogeneous spatial distribution of MXene in the matrix as evidenced by the titanium element. Furthermore, the EDX mapping is shown for the as‐fabricated composite and a sample after 15 months of repeated cycling in acetone and humid atmosphere with prolonged storage under ambient cleanroom conditions in between. The extracted weight and atomic percentage values of the relevant elements, carbon, oxygen, and titanium, indicate a substantial oxidation of the material since the amount of oxygen has doubled while the other two elements remain almost constant. This is similar to the results of the pure MXene (Figure 2), but in that case, the strong oxidation was already evident after six weeks, with the material becoming unresponsive in the repeated chemiresistive testing (Figure 1). In contrast, the composite remained functional for at least three months, as shown in **Figure** **5**.

#### 2.3.3 Chemiresistive IDE Analysis

Figure 3c,d depict optical microscope and SEM images of the fabricated composite material on IDEs and the corresponding chemiresistive measurements in varying environmental conditions. As in the case of pure MXene, air‐dried and freeze‐dried samples have been studied. It is clearly evident that the drying method exerts a significant influence on the sample structure as described in the microstructural comparison above. This evident difference in structure is further reflected in the gas sensing performance of the composite material. As depicted in Figure 3c, the limited surface area of the air‐dried composite results in a constant resistance with no response to additional acetone molecules. An initial increase in resistance is observed up to ≈50 min and then stabilizes upon reaching equilibrium.

In contrast, the freeze‐dried MXene/PNIPAAm composite exhibits a distinct response depending on the gaseous environment, and it furthermore shows an overall significantly increased resistance value of ≈100 MΩ compared to the air‐dried counterpart (Figure 3d). This can likely be attributed to the highly porous structure, which leads to a larger spacing in between the embedded MXene sheets.

With the introduction of saturated water vapor (first as dynamic flow to clean the chamber and then in the static environment with the flow turned off), a decrease in resistance occurs due to the interaction of water molecules with the sample. Upon exposure to gaseous acetone, a sharp drop in resistance is observed (indicated by red arrows), followed by a further decrease but with a much smaller slope.

When the chamber is then purged with water vapor only, the resistance immediately starts to increase again in the vapor flow and decreases in the static water vapor condition (flow turned off). This curve shape is consistently observed, but the magnitude of the initial drop and the subsequent downward slope due to the introduction of acetone are dependent on the acetone concentration. A hypothesis for explaining these observations in response to acetone is provided in the discussion Section 2.4.3.

Overall, the magnitude of the initial resistance drop and the response time, i.e., the slope of the curve, are dependent on the acetone concentration. A higher concentration results in a larger resistance change and longer response time. The corresponding response times t_90_ and magnitudes of the initial resistance change for the acetone response of the freeze‐dried composite are listed in Table S1 (Supporting Information). When comparing the sharp drop induced by exposure to an acetone atmosphere with existing research on polymer‐based composite materials for acetone sensing, the calculated response times are in a very similar range.^[^ ^46^, ^59^ ^]^

Another observation that is made from the first three cycles of the same acetone concentration (100 ppm) is a settling effect of the composite material. This is attributed to the almost inevitable conditioning process characteristic of hydrogel sensing materials after preparation or introduction into a new environmental setting.^[^ ^60^ ^]^ During this phase, the first few cycles of swelling/deswelling with or without the stimulus cause microscopic changes and settling processes in the polymer network. These can result in an initially reduced repeatability and drift of the sensor response. However, through repeated cycling under the same conditions, the repeatability is stabilized, which is the case for the studied composite.

The observed sample reaction to the water vapor flow purging can be explained as follows: Opening the inlet leads to a significant increase in water molecules in the chamber that compete with the acetone molecules in the composite, fostering a removal of the organic solvent, and resulting in an increase in resistance. When the flow is turned off after 10 min and the sample is left to settle again in the static humidity condition for another 10 min, more water molecules can be adsorbed on the surface, leading to a swelling change and an altered conductivity. Depending on the microstructure of the composite (2D or 3D, i.e., air‐ or freeze‐dried, respectively), either one effect can be more pronounced. In the 2D case, the swelling/deswelling of the hydrogel component alters the spacing between the MXene parts, which ultimately causes the change of resistance (i.e., swollen hydrogel – larger spacing – higher resistance, and vice versa). For the 3D structure, the conductivity increasing effect appears to be more significant as reflected by the slight decrease in resistance.^[^ ^61^ ^]^

The 10 min time interval for purging has been chosen based on the ratio of chamber volume and amount of injected acetone and is deemed sufficient for the complete removal of any organic solvent residue (also based on previous work^[^ ^39^ ^]^).

#### 2.3.4 Repeated Chemiresistive Analysis

To evaluate the longer‐term stability of the composite and the occurrence of degradation effects, multiple IDEs equipped with the MXene/PNIPAAm composite were repeatedly tested under the same conditions: cycling of acetone from 20 to 100 ppm and back in water vapor atmosphere (≈160 min for one full cycle), storage for 1 week under cleanroom conditions (22 °C, 45% RH, open box) and then retesting.

Please note that for this study, soft IDEs based on polyimide and with platinum metallization have been used (refer to Figure S8, Supporting Information for the IDE layout). The reason for this was that it proved difficult to obtain a stable adhesion of the composite to the rigid IDEs for prolonged amounts of time. The samples tended to fall off after synthesis, which was attributed to the mechanical mismatch at the interface between the glass‐/ceramics‐based IDEs and the comparatively soft MXene/PNIPAAm composite. By using a softer base material for the IDE, a larger number of intact samples could be obtained. This challenge only occurred for the composite material, but not for the pure MXene.

Figure 5 depicts the IDE's delta resistance for each acetone condition and repeated testing. The delta resistance is calculated based on the sharp drop observed in the curves (Figure S11, Supporting Information). Please note that the first test, which was used to condition the polymer material in an acetone gas atmosphere to obtain a more stable output, is excluded.

From the diagram in Figure 5, it is evident that the sample exhibited an increased response to acetone in the third and fourth tests. However, in the fifth test, it dropped to a level similar to that of the second test. Nevertheless, compared to the second test, where the material showed residual gas (indicated by an increased delta resistance at the same acetone concentration), the output in the fifth test demonstrated better stability and reproducibility.

#### 2.3.5 Mechanical Properties

**Figure 6:**
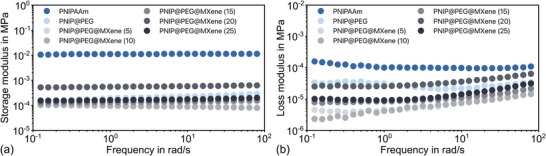
Rheological test results of pure PNIPAAm and MXene/PNIPAAm composite samples with different MXene concentrations in the range of (5–25) mg mL^−1^ in the suspension (indicated by numbers next to the composite type in the legend): a) storage and b) loss modulus in frequency scan mode. Angular frequency scan from (0.1 to 100) rad s^−1^.

To investigate the influence of the MXene on the mechanical properties of the composite, corresponding bulk samples with varying MXene concentrations (ranging from (2 to 25) mg mL^−1^ in the original suspension) were characterized by rheology (**Figure** **6**). Pure PNIPAAm hydrogel with and without PEG was also included for comparison. Furthermore, all samples were tested in the watery swollen state.

Pure PNIPAAm without any additives or porogen shows the highest values for both storage and loss moduli. By creating pores through the addition of PEG, the moduli are reduced and decline even further when MXene is added in low concentrations. This indicates a reduction of cross‐linking strength.^[^ ^62^ ^]^ However, with increasing MXene concentration (25 mg mL^−1^ and higher), the material becomes tougher again, with the storage and loss moduli reaching similar or even slightly higher values than the PEG‐modified PNIPAAm.

In all cases, the storage modulus is always larger than the loss modulus, indicating the chemical cross‐linking and solid‐like behavior of the material. Furthermore, the storage moduli are almost independent of the oscillation frequency, while the loss moduli exhibit a more pronounced frequency dependence for all modifications (PEG alone and all MXene concentrations). This indicates an elastic deformation of the samples.

### 2.4 Discussion

#### 2.4.1 Comparison of Mechanical Properties of Pure MXene and MXene/PNIPAAm Composite

As shown in Figure 6, the pure PNIPAAm hydrogel without added PEG for pore generation has the highest storage and loss moduli, and both values are substantially reduced when the porogen is used. The incorporation of low concentrations of MXene in the composite reduces the mechanical stability even further, since it affects the cross‐linking density during polymerization. An increased MXene content yet improves the mechanical properties.

A similar phenomenon has been reported in other research, showing that relatively low and high MXene contents can produce transient structures dominated by MXene or by polymers, with higher yield stresses.^[^ ^63^ ^]^ For structures containing MXene that lie in between, the lower filler content could result in a reduced crosslinking degree of the composite hydrogels.^[^ ^62^ ^]^

A further increase in MXene concentration will lead to the development of electrostatic interactions between the polar groups of the MXene nanosheets and the functional groups of the polymer chains, expanding the number of physical crosslinking points and ultimately improving the mechanical properties.^[^ ^60^ ^]^ However, an excess of MXene in the polymer matrix leads to an increased viscosity of the precursor, making it challenging to fabricate intact samples of larger size. Furthermore, it can promote agglomeration and hinder the polymerization process. In our case, we found it impossible to create intact composite samples for MXene concentrations larger than 30 mg mL^−1^. In summary, the MXene content can be used to tailor the mechanical properties of the MXene/PNIPPAm composite, but needs to be carefully balanced with the precursor recipe and the amount of porogen.

#### 2.4.2 The Role of the Porogen

The primary purpose of using a porogen with a hydrogel is the creation of an adjustable porous polymer network (bulk and surface). Our results for the MXene/PNIPAAm composite reveal that the porogen (here long‐chain PEG) is also crucial for achieving a homogeneous dispersion of MXene in the hydrogel during polymerization. As depicted in Figure S9a (Supporting Information), the MXene/PNIPAAm composite synthesized by a simple mixture of monomer precursor and MXene suspension yields a heterogeneous structure after freeze‐drying. The optical microscope image indicates a visible black‐and‐white pattern on the sample surface, while SEM images show dimpled surfaces of the composite. In addition, the sample exhibits an inhomogeneous combination of dense and porous structures in the cross‐section. Previous work has shown that this indicates a high risk for pore collapse, i.e., low structural integrity.^[^ ^39^ ^]^ The EDX mapping results (Figure S10a, Supporting Information) further validate this phenomenon: the partially collapsed structure of the plain MXene/PNIPAAm composite shows a pronounced accumulation of titanium.

A much more homogeneous MXene distribution within the composite is achieved by utilizing PEG during the polymerization. The combination of SEM and EDX analysis in Figures 3 and 4 evidences the homogeneous integration of MXene within the polymer matrix. Furthermore, the microstructure resembles a PEG‐modified PNIPAAm sample without any filler (Figure S6, Supporting Information), highlighting the predominance and scaffolding role of the polymer in this composite.^[^ ^39^ ^]^ No individual MXene sheets can be identified in the SEM images, and the EDX results (Figures 3, 4; Figure S9b, Supporting Information) clearly confirm a uniform titanium distribution.

These results highlight the importance of the interplay between the hydrogel and porogen for the creation of a 3D MXene arrangement. While the polymer assumes the scaffolding role, it is the spatial hindrance caused by the long‐chain polymer molecules during composite synthesis that ultimately leads to a homogeneous MXene distribution.^[^ ^65^ ^]^

On one hand, the presence of PEG induces a phase separation of PNIPAAm during synthesis, leading to the formation of a macroporous hydrogel structure.^[^ ^66^ ^]^ On the other hand, PEG creates steric hindrance in the crosslinking process, leaving behind inherent voids in the resulting polymer matrix after particle leaching.^[^ ^66^ ^]^ This spatial constraint prevents the aggregation of MXene sheets into their usual stacked structure during the polymerization process.

Consequently, the use of a porogen (PEG in the presented case) is crucial for achieving a uniform dispersion of MXene flakes within the composite. This finding opens up the possibility of creating controlled porosity and spatial MXene distribution by the choice of the porogen (material type, molecular weight, amount) and the freezing temperature during synthesis. Moreover, with the presented synthesis process, the porous structure of the composite remains stable in gaseous environments with varying relative humidity, as previously demonstrated^[^ ^39^ ^]^ due to the scaffolding role of the polymer.

However, it is imperative to strike a balance between monomer concentration and porogen content to achieve an intact composite material. An increase in porogen may result in unsuccessful polymerization if the monomer concentration is too low in comparison.

#### 2.4.3 Chemiresistive Sensing Analysis

In the chemiresistive sensing setup, a current is applied to the IDE, which passes through the sample placed on the electrode structure. The measured output resistance is proportional to the sample's conductivity, which depends on the material properties as well as external influences such as adsorbed gas molecules. Consequently, the method can be used to detect gases and their concentration with a known sensing material, as well as for the characterization and comparison of different sensing materials (by using the same gas and concentration).

In the presented study, the chemiresistive analysis was used with the test organic gas acetone, to evaluate the response and deduce material properties of the pure air‐dried and freeze‐dried MXene and the MXene/PNIPAAm composite.

To assess the experimental results of the pure and composite samples, first, a phenomenological description of the underlying processes occurring in the MXene component is provided. This is based on the comprehensive analysis of Pazniak et al. and Sunderiya et al., who have investigated the conductivity mechanisms and responsiveness to organic gases of Ti_3_C_2_T_x_ MXene in its pristine and partially oxidized state^[^ ^23^ ^]^ and the oxidation progression of Ti_3_C_2_T_x_ MXene in aqueous solution.^[^ ^67^ ^]^

**Figure 7:**
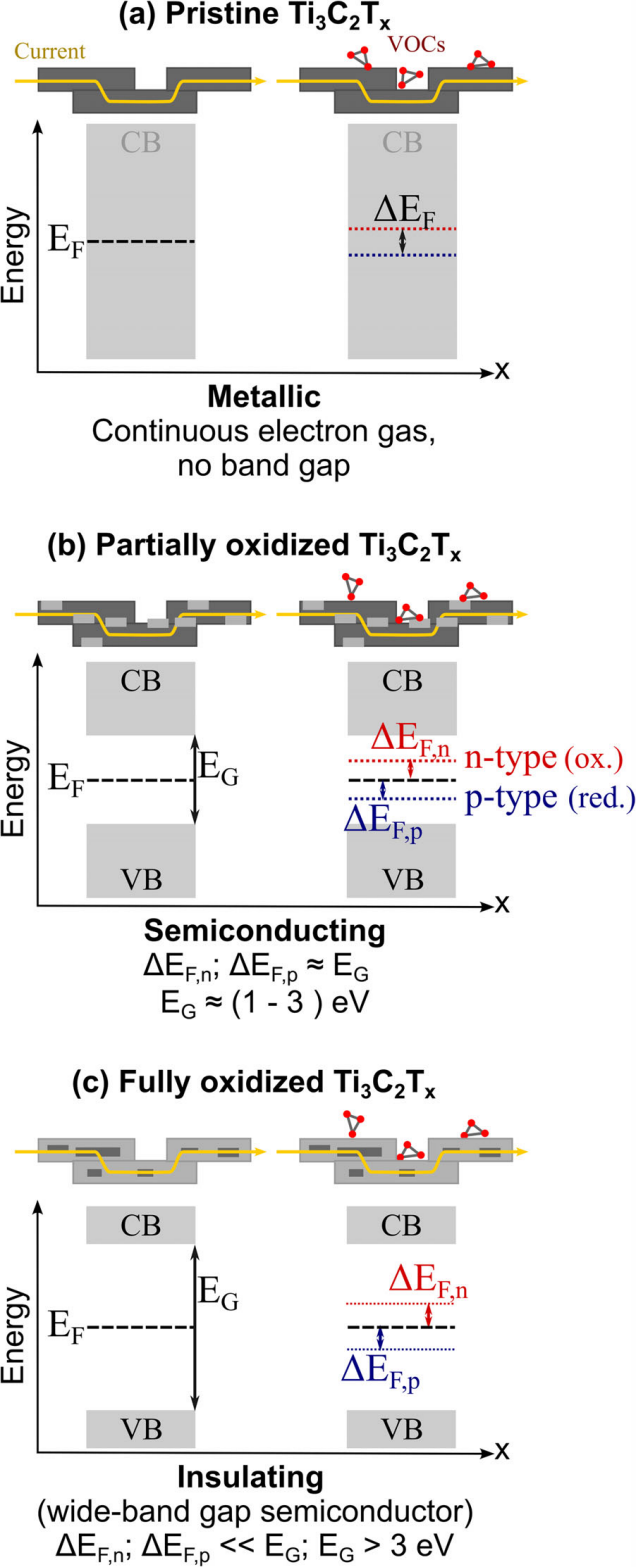
Simplified principle of the conduction behavior of (a) pristine, (b) partially oxidized, and (c) fully oxidized Ti_3_C_2_T_x_ MXene. E_F_, ΔE_F,n,_ and ΔE_F,p_ denote the Fermi energy and its shift toward the conduction (CB) or valence (VB) band in the presence of oxidizing or reducing gases, respectively. E_G_ denotes the height of the energy barrier between the bands. In each diagram, the left and right parts depict the energy band structure without and with MXene, respectively. The sketches above the energy diagrams illustrate the current passing through the linked MXene sheets, the VOC adsorption, and the oxidation state (dark grey: pristine MXene, light grey: TiO_2_). The described concept is based on investigations published in refs. [23, 67].

Applying the insights from the aforementioned studies to the presented chemiresistive investigations leads to the following conceptual understanding of MXene‐based samples on IDEs (see **Figure** **7**). In its as‐fabricated state, Ti_3_C_2_T_x_ MXene exhibits metallic conductivity, similar to a 2D electron gas, allowing current to pass through interconnected nanosheets with low resistance (Figure 7a). Gas adsorption on the MXene surface introduces additional charge carriers, but their effect is negligible due to the intrinsic metallic conductivity. Instead, the adsorbed molecules act as scattering centers, increasing overall resistance. Hence, pristine MXene always shows a resistance increase upon gas adsorption, independent of gas species.

Oxidation of the titanium component fundamentally changes this behavior. TiO_2_, a wide‐bandgap semiconductor,^[^ ^68^ ^]^ causes the material to shift from metallic to semiconducting and eventually insulating states. Partial oxidation forms Schottky‐type heterojunctions within and between MXene sheets, transforming the continuous electron gas into a band structure with an energy barrier. This barrier is small enough that gas‐induced charge carriers increase conductivity, acting like donor or acceptor dopants.^[^ ^59^ ^]^ As shown in Figure 7b, oxidizing gases shift the Fermi level toward the conduction band, and reducing gases toward the valence band, hence both lower the barrier height and thus reduce resistance. However, the total resistance remains higher than that of pristine MXene due to the numerous heterojunctions.

With further oxidation, the bandgap widens, and the energy barrier becomes too large for gas‐induced charge changes to affect conductivity. The material then reaches very high resistance, becoming unmeasurable by chemiresistive methods. Based on this model, the chemiresistive behavior of 2D (air‐dried) and 3D (freeze‐dried) MXene samples and their composites is discussed in the following.

##### 2.4.3.1 Pure MXene

According to the results of the chemiresistive response to varying acetone concentrations depicted in Figure 1c, pure 2D MXene exhibits an increase in resistance independent of the acetone concentration, with an overall resistance of a few hundred Ohms. This is consistent with a metallic conductivity behavior and the findings of other publications.^[^ ^3^, ^56^, ^69^ ^]^

In contrast, the results for the 3D MXene structure depicted in Figure 1d indicate a semiconducting response since the resistance decreases in the presence of acetone, while the overall resistance is much larger (Megaohms) compared to the 2D sample. It can be assumed that this change in conductivity behavior is due to the highly porous structure of the material visible in the SEM images. The substantially increased surface area compared to 2D MXene fosters oxidation and, thus, the transition into the semiconducting state. The progression of the oxidation is furthermore evident in the repeated testing of the sample with the same acetone concentrations (Figure 1d), where the resistance kept increasing over six weeks until it became immeasurable (insulating state). In addition, the results of the four‐week sample test show a clear dependence of the resistance decrease on the acetone concentration for the range of (20–80) ppm. For 100 ppm the decrease is reduced, which is attributed to a saturation effect of the material.

It needs to be noted here that the chemiresistive analysis of all pure MXene samples has been conducted with a dried nitrogen background instead of air. It is thus expected that the material would have degraded and lost its responsiveness even earlier if a humid background similar to that of the composite had been used.

The oxidation of the titanium in the MXene occurs randomly and rather uncontrolled. However, our findings indicate that one potential way to exert at least some influence on the progression of oxidation is by controlling the porosity of the surface and bulk parts of the material, as well as the pore size, by the freezing temperature and drying method.

##### 2.4.3.2 MXene/PNIPAAm Composite

The chemiresistive response of the composite material is more complex as both the MXene and the PNIPAAm hydrogel respond to the acetone gas, and, in particular, the hydrogel also responds to the humid air. It needs to be noted that the humidity is necessary to create a measurable output resistance. With a dried nitrogen background, as in the case of pure MXene, no output signal could be obtained. It is therefore assumed that the polymer at least partially passivates the MXene and reduces the contact between individual sheets. Such a passivation behavior has, for example, been reported for a combination of polydopamine (PDA) on MXene.^[^ ^51^ ^]^

In its fully dried state, the composite is non‐conductive, but the presence of water molecules enables current pathways through the material.

Comparing the chemiresistive results of the 2D (air‐dried) and 3D (freeze‐dried) composite depicted in Figures 3c and 4d, it is clearly evident that the 2D sample does not show any detectable resistance change to varying acetone concentrations. It only undergoes a conditioning process in the first two cycles, where the resistance is slightly increasing, likely due to the relaxation and rearrangement of the polymer chains in response to the higher relative humidity (RH = 100%) in the test chamber compared to the cleanroom storage conditions (RH = 45%). This behavior can be attributed to the non‐porous and skin‐like sample surface visible in the SEM images. This likely prevents water and gas molecules from penetrating the material body and initiating a response.

In contrast, the very open and interconnected porous structure obtained by freeze‐drying creates a very large surface area for the interaction with water molecules from the humid air background, as well as the adsorption of acetone gas molecules. The observed response to the presence of acetone (Figure 3d) can be attributed to two effects: i) the interaction of MXene with acetone, and ii) the physical swelling response of the hydrogel that, in itself, would cause an increase in resistance. These two effects exhibit different time constants. While the charge transfer interaction between MXene and the gas molecules happens very fast, the hydrogel swelling is a diffusion‐based process that relies on the analyte being adsorbed on the surface and subsequently reaching the inner part of the polymer network. This causes a comparatively slow response until the hydrogel reaches a swelling equilibrium. Considering the curve shape of the acetone response depicted in Figure 3d, it can be assumed that the initial sharp drop in resistance is caused by effect (i), since it is consistent with the conductivity behavior of partially oxidized MXene.

Once the hydrogel starts to swell, the effect is partly counteracted by the increasing distance between the MXene flakes embedded in the polymer. This likely leads to the second part of the curve, where the resistance keeps decreasing but with a substantially reduced slope compared to the initial drop. Whether the step height of the initial drop or the slope of the slowly decreasing resistance is more informative in terms of a sensing application for acetone needs to be evaluated in further studies.

The overall resistance of the 3D composite sample is comparable to the pure 3D MXene, indicating that the base conductivity is determined by the partially oxidized MXene and not the hydrogel or the presence of water molecules.

#### 2.4.4 Degradation Stability

The oxidant responsible for the MXene transition from metallic conductivity to semiconducting and subsequently insulating is the oxygen from air and water molecules.^[^ ^67^ ^]^ During sample storage under cleanroom conditions, this non‐specific oxidation progresses over time is evidenced by the changing chemiresistive response.

Furthermore, the specific experimental conditions (acetone exposure, high humidity in the case of the composite material) during the chemiresistive measurement itself may also contribute to the degradation. However, the duration of one full‐cycle experiment that covers different acetone concentrations is only 160 min, which is negligible compared to the constant exposure to humid air during weeks of sample storage.

By comparing the degradation progression of the 3D pure MXene and composite sample, the role of the hydrogel can be deduced. In both cases, the highly porous 3D microstructure fosters the initiation of the oxidation processes right after sample fabrication. For 3D pure MXene, the oxidation progresses continuously until the output resistance becomes immeasurably high within 6 weeks after fabrication. In contrast, repeated chemiresistive cycling of the composite (Figure 5) shows that the resistance change first becomes larger with retesting (tests 3 and 4) and then starts to decrease again (test 5) over a period of three months (almost 13 weeks). This indicates a passivation effect of the PNIPAAm hydrogel and a substantially prolonged oxidation stability compared to pure 3D MXene. However, 15 months after fabrication, the composite sample is fully oxidized and immeasurable as well. This is corroborated by the EDX results for both the pure 3D MXene and composite samples, which indicate a similar doubling of the oxygen content (Figures 2 and 4).

These initial investigations indicate the potential of the studied MXene/PNIPAAm composite with respect to VOC sensing, but also for an advanced future material design where the properties of both components are combined to create new functionalities. Our study shows that the combination of the developed porosity engineering techniques for polymer materials, such as the use of a porogen, variation of drying method, and freezing temperature, can substantially alter the microstructural properties and spatial distribution of MXene in a hydrogel composite, and consequently its degradation stability and conductivity behavior. The creation of electronic states is affected by the interplay between polymer and MXene, which furthermore reflects on the potential usability of the composite as a gas‐sensing material.

## 3 Conclusion

This work presents a fundamental investigation of the properties of a novel dry MXene/PNIPAAm composite and its potential for an application in volatile organic compound (VOC) detection in gaseous environments using chemiresistive transduction. Therefore, pure MXene and composite samples featuring either a compact and dense or a highly porous 3D microstructure have been fabricated and characterized. These investigations reveal advanced properties with respect to MXene degradation stability and control over the 3D structural arrangement.

With regard to electronic properties, the results show that the tailored porosity of pure MXene, namely from a densely packed to a fully 3D highly porous microstructure, allows for alteration from metallic to semiconducting behavior in response to a VOC interaction in a chemiresistive sensing configuration. This is due to the oxidation of the titanium in MXene being dependent on, and therefore adjustable by, the porosity. Furthermore, the creation of partially oxidized MXene is simply achieved through the tailoring of the 3D structure without the need for the commonly used additional heating.

The oxidation of pure 3D MXene under ambient conditions occurs rather rapidly and uncontrolled, leading to the loss of conductivity and responsiveness to gas interactions within four to six weeks. In contrast, the composite exhibits a significantly slowed degradation behavior. It maintained its responsiveness and semiconducting state for at least 12 weeks under repeated testing in the same variations in acetone levels in a range of 20 ppm to 100 ppm, with storage under ambient cleanroom conditions in between tests. This is attributed to a passivation influence of and interplay with the polymer.

The underlying mechanisms of this discovery need to be studied further. So far, two major implications can be derived: i) The composite significantly reduces the speed of the oxidation‐induced degradation of the MXene, thereby stabilizing the electronic state and interaction with gas species. ii) The porosity characteristics (e.g., pore distribution, size, and interconnection) enable control and adjustment of the composite's electronic state and sensitivity to gas interactions. On the one hand, a larger number of pores can increase the resistance. On the other hand, it also creates a larger surface area for the oxidation of the titanium and consequently, the formation of Schottky‐heterojunctions that increase the responsiveness to gas adsorption.

The hydrogel provides a 3D scaffold into which the MXene is fully integrated without the usual stacking of sheets. The microstructure, spatial MXene arrangement, and porosity of the composite can be tailored by the choice of the porogen, the drying method (air‐ or freeze‐drying), and the freeze‐drying temperature. Thus, the application of the porosity engineering techniques developed for the polymer enables fundamentally novel avenues for tuning and tailoring MXene properties with the integration into a composite material.

From the perspective of a sensing application for VOC detection, the control of the interplay between the polymer and MXene through the microstructure can be employed to adjust the analyte sensitivity from almost non‐responsive (for a dense and compact microstructure) to strongly responsive (for a 3D porous). In the latter case, the distinctly different interaction mechanisms of charge transfer of the MXene and mechanical swelling/deswelling of the hydrogel with the test analyte acetone are reflected in a very distinct resistance change pattern. In the context of gas sensing, this enables: i) a high sensitivity to acetone concentrations as low as 20 ppm, ii) a stable and reproducible response, and iii) the potential for very fast tracking of changes in analyte concentration (within a timeframe of less than 2 min).

Moreover, the described techniques for the fabrication of a tailored microstructure of the composite are of a general nature and not specific to the PNIPAAm hydrogel used in this study. Hence, they can be applied to any other type of polymer and its integration with MXene, thereby enabling the development of sensing applications for a wide range of analytes due to the versatility of the polymer.

From the overall perspective of advancing material science, the presented study demonstrates the creation of a composite by merging the well‐known class of polymers with the novel group of MXenes. By employing techniques originally developed for the porosity engineering of hydrogels, the resulting material features a complex interplay between both constituents that offers great potential for addressing some of the remaining major challenges associated with MXene use to date, namely oxidation‐induced material degradation, control over electronic state, and creation of a fully 3D microstructure. Furthermore, it lays the foundation for applications in gas sensing that utilize the stimulus‐interaction capabilities and biocompatibility of hydrogels with the electronic properties enabled by MXene.

## Conflict of Interest

The authors declare no conflict of interest.

## Supporting information



Supporting Information

## Data Availability

The data that support the findings of this study are available from the corresponding author upon reasonable request.
