# Supplementary material for: A 3D Porous MXene/PNIPAAm Hydrogel Composite with Advanced Degradation Stability and Control of Electronic Properties in Air
Source: Adv Sci (Weinh). 2025 Nov 14;13(6):e16529. doi: 10.1002/advs.202516529 (PMC12866718; doi:10.1002/advs.202516529)
Supplement: Supplementary file 1 — Supporting Information [file ADVS-13-e16529-s001.pdf]

A 3D porous MXene/PNIPAAm hydrogel composite with advanced degradation stability and control of electronic properties in air

Sitao Wang [b]#, Chen Jiao[c], Gerald Gerlach[b] and Julia Körner*[a]

[a] J. Körner
Department of Electrical Engineering and Computer Science
Leibniz University Hannover
Schhneiderberg 32, 30167 Hannover, Germany
E-mail: koerner@mbe.uni-hannover.de

[b] S. Wang; G. Gerlach
Institute of Solid-State Electronics
Dresden University of Technology
Helmholtzstraße 10, 01069 Dresden, Germany

[c] C. Jiao
Leibniz Institute of Polymer Research Dresden
Hohe Straße 6, 01069 Dresden, Germany

#The presented research was conducted under affiliation [b], but Sitao Wang's current affiliation is [c].

Content

1. Sample fabrication and processing
2. Sample characterization
3. Supporting figures
4. Supporting tables
5. References

1. Sample Fabrication and Processing

Overview

In all cases where MXene was used, a corresponding suspension of a concentration of 30 mg/mL was first made from powder and then used for further processing. This concentration was chosen based on prior experiments, which showed that 30 mg/mL is the highest MXene content that still enables a successful polymerization in combination with the target hydrogel PNIPAAm. Higher MXene contents cause incomplete polymerization and subsequent disintegration of the samples. Lower MXene concentrations are studied in the context of mechanical properties of the composite as described in section 3(e) in the main text.

The 3D pure MXene network was obtained by freezing-induced preassembly of the MXene suspension followed by nanosheets cross-linking in hydrochloric acid (HCl). The former offers a tunable microstructure, where the formation of ice crystals plays a crucial role in determining the final structure.[S1,S2] Therefore, two different freezing temperatures (-196 °C and -20 °C) were studied. The latter ensures a reinforced scaffold by intercalating cations between the sheets at a high concentration, resulting in three-dimensional interlinking of 2D MXene.[S3] The 3D MXene structures were fabricated on IDEs with platinum metallization by drop-casting.

The MXene/PNIPAAm composite was created by mixing the monomer precursor with the prefabricated MXene suspension. Additionally, long-chain poly(ethylene glycol) (PEG) was included as a pore-forming agent as described in previous work.[S5] For the presented study, PEG of a molecular weight of 10,000 was used (FLUKA, Germany, used as received). It creates intrinsic voids during polymerization, resulting in a large surface area of the polymer after PEG removal. The quantity of the applied porogen must be sufficient to create adequate voids in the matrix without exceeding a threshold that disrupts the cross-linking of the entire sample, potentially causing polymerization failure. To align the mechanical properties of the material with the IDE substrate and ensure good adhesion between them, IDEs made of platinum on a flexible polyimide substrate and gold on a ceramic substrate were utilized.

WILEY • VCH

To stabilize the porous microstructure of the fabricated materials for the intended use of gas sensing, post-fabrication processing includes the steps of washing (to remove all unreacted chemicals), freeze-drying in liquid nitrogen and, only in case of the hydrogel-containing samples, subsequent conditioning in high humidity to allow the polymer chains to reconfigure to an equilibrium state. This procedure has been previously developed and demonstrated to enable a stable porous microstructure of PNIPAAm hydrogel in gaseous environments of varying relative humidity and for VOC sensing.[S5] Due to the reduced sample size and the improved mechanical properties of the MXene/PNIPAAm composite, in the presented experiments, the first test in the organic/humid gas atmosphere was used to stabilize the material structure, rather than conditioning the sample in high humidity for 3 days. This approach suffices to ensure reproducible results in the subsequent testing within the same gaseous environment.

Detailed Fabrication Processes

Bulk pure MXene structure: 3D MXene structures were fabricated by freezing-induced preassembly and a subsequent treatment in protic acids.[S1,S3] By shifting their surface zeta potential in protic acids like hydrochloric acid (HCl) and sulfuric acid (H_2SO_4), negatively charged MXene nanosheets cross-link and form a 3D structure due to the electrostatic interaction. These obtained 3D scaffolds are also commonly denoted as MXene hydrogels. In the presented case, $Ti_3C_2T_x$ powder was purchased from Nanoplexus Ltd. (England, UK) and used directly without further purification. To obtain a homogeneous MXene suspension, 30 mg powder was dispersed in 1 mL deionized water and sonicated in an ice bath for 3 h. Then the $Ti_3C_2T_x$ dispersion was poured into glass molds with a 1200 µm thick PTFE spacer. The mold edges were secured with binder clips (19 mm) to prevent leaking of liquid. For ice templating, the molds were either placed in liquid nitrogen (-196 °C) for 5 min and subsequently transferred to a refrigerator (-20 °C) or kept directly in the refrigerator for 24 h. Afterwards, the frozen sample was put into 12 M HCl (37%, VWR, Germany) solution and stored for another 24 h. The acid solution was then replaced with deionized water for 3 days with daily solution change until the acid had been completely removed. Ultimately, to preserve the porous structure, the samples were freeze-dried with liquid nitrogen (-196°C).[S4,S5]

Pure MXene structure on IDE: Due to the fragile nature of the porous pure MXene structure, using a mold system with a thinner spacer is not feasible. Therefore, a simple drop-casting method was employed (Figure S1a). In this method, a hemispherical droplet was manually pipetted onto the IDE surface (glass substrate), consisting of 8 µL of MXene suspension. Subsequently, the entire IDE with the deposited material on top was either air- or freeze-dried as described below.

Bulk MXene/PNIPAAm composite with PEG porogen: The MXene/PNIPAAm composites were prepared by simply mixing the hydrogel with the MXene suspension. PEG was employed as a pore-forming agent, which was removed after polymerization by washing in deionized water for 7 days with daily liquid exchange. Specifically, 1 mL of MXene suspension at a concentration of 30 mg/mL was mixed with 1 mL of NIPAAm precursor containing 1 g of PEG.
To prepare the hydrogel precursor, NIPAAm (4.42 mmol, 0.5 g) and MBA (0.21 mmol, 0.033 g) were fully dissolved in 3 mL of deionized water and degassed with nitrogen for 5 min. Then, TEMED (0.05 mmol, 7.5 µL) and APS (0.07 mmol, 0.0167 g) were added, followed by 1g of PEG as porogen. After 3 days of polymerization in a cleanroom environment and leaching of PEG in deionized water for another 3 days, the sample was either freeze-dried by freezing in liquid nitrogen (-196 °C) or air-dried. For more details on the utilization of PEG, please refer to our previous work.[S5]

MXene/PNIPAAm composite with PEG porogen on IDE: Due to the mechanical flexibility of polymers, it is possible to achieve precise control over the composite thickness and shape with a mold in case of the MXene/PNIPAAm on IDEs (in contrast to pure MXene). The IDE was initially placed within a mold system, where a Teflon spacer of a defined thickness (100 µm) was positioned between two glass slides (Figure S1b). The precursor solution was then injected into the space between one of the glass sheets and the IDE surface, resulting in the successful fabrication of a thin layer of MXene/PNIPAAm composite on top of the IDE. Following a 3-day polymerization period, the desired material shape (full coverage of the IDE electrode structure) was obtained by carefully cutting it with a blade. The leaching of PEG, as previously described, was continued for another 3 days by submerging of the IDE sample in deionized water with daily liquid exchange. Finally, the sample was either freeze- or air-dried.

Freeze-drying of fabricated hydrogel-containing samples: After being fully rinsed in deionized water (resulting in a swollen state), the samples were transferred into 50 mL glass vials compatible with the connectors of the freeze-dryer. There were two options for freezing: i) immersion of the samples in liquid nitrogen (-196 °C) for 10 min, followed by transfer to the freeze-dryer, which maintained reduced pressure for the gradual sublimation of ice; or

WILEY-VCH

ii) placement of the samples in a freezer (-20 °C) for 24 h before moving them to the freeze-dryer. In both cases, all samples were connected to the drying equipment for a continuous 24 h period to ensure the complete sublimation of ice crystals.

Air-drying of fabricated hydrogel-containing samples: After polymerization and rinsing for 7 days in DI water, the swollen sample was removed from the water and left to dry in open air under cleanroom conditions (22 °C, 45% RH) for 3 days. Based on previous studies, this ensures complete removal of liquid.[S4,S5]

Materials and Chemicals

N-isopropylacrylamide (NIPAAm), N,N'-methylenebis(acrylamide) (MBA), ammonium persulfate (APS), N,N,N',N'-tetramethyethy-lenediamine (TEMED), lithium phenyl-2,4,6-trimethylbenzoylphosphinate (LAP) were purchased from Sigma-Aldrich (Germany) and were used as received. Poly(ethylene glycol) (PEG) with molecular weight of 10000 was purchased from FLUKA (Switzerland) and used as supplied. MXene ($Ti_3C_2T_x$) powder was purchased from Nanoplexus Ltd. (United Kingdom) and used directly without further purification. 37% hydrochloric acid (HCl) solution was obtained from VWR (Germany).

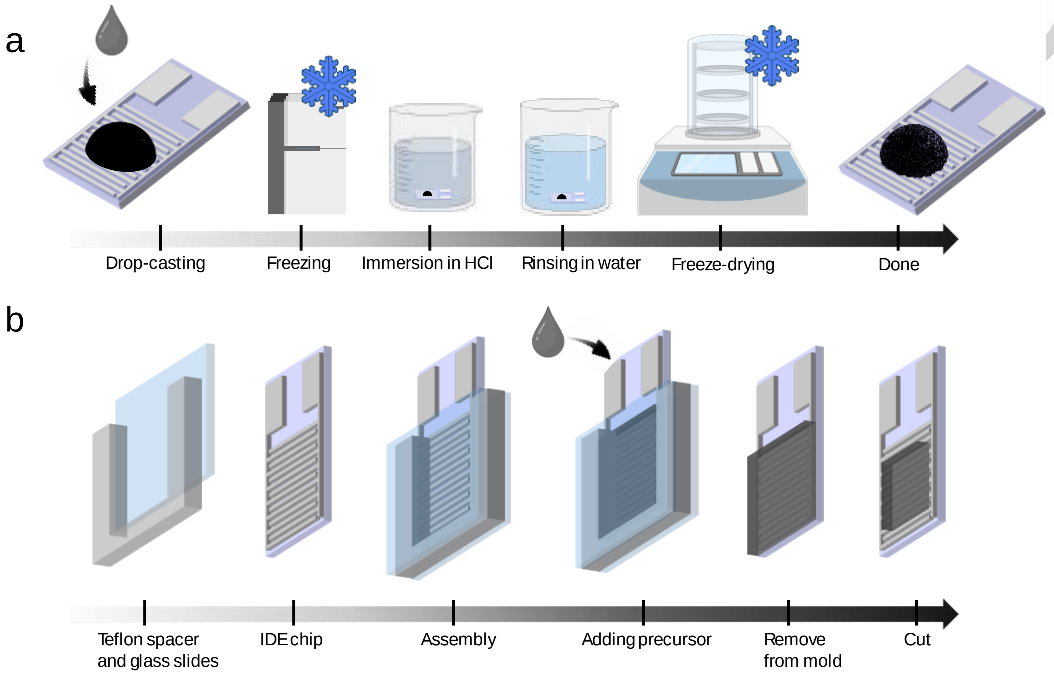

Figure S1. Fabrication process of (a) pure MXene and b) MXene/PNIPAAm composite samples on IDE.

2. Sample Characterization

Appearance and Microstructure

Optical microscope images were taken with a stereo microscope (Leica MZ6, Switzerland) with lighting (SHOTT KL 750, Germany). The detailed surface morphology and internal structure of the materials were characterized by using a Zeiss Supra 40 VPF scanning electron microscope (Carl Zeiss GmbH, Germany). For hydrogel-based materials, the samples had to be dehydrated and coated with 10 nm of gold to avoid charging effects due to the low electrical conductivity of the polymers. Gold deposition was performed by sputter coating (SC7620 Mini Sputter Coater, Polaron, Germany). For 3D MXene structures, no gold deposition was required. Additionally, EDX (EDAX, ELECT PLUS, AMETEK, USA) was employed to study the elemental distribution in the samples. Samples were prepared in the same manner as for SEM.

Mechanical Properties

Hydrogel-based bulk samples were assessed by rheology using a strain-controlled rheometer (ARES-G2, TA Instruments, USA) with a parallel plate geometry of 25 mm in diameter. With a fixed oscillatory strain of 1%, a dynamic frequency sweep in the range from 0.1 to 100 rad/s was conducted at 22 °C.

Chemiresistive Performance

To investigate the chemiresistive response of the sensing materials to varying levels of acetone, the chip was placed in a sealed chamber where the desired environmental conditions could be produced by injecting a defined volume of liquid organic solvent. The general setup is depicted in figure S2. The acetone concentration in the chamber was adjusted from 20 to 100 ppm, with steps of 20 ppm in between and the calculation details are outlined in figure S3.
Predefined humidity conditions were created by placing two beakers of 1 mL of deionized water in the sealed chamber which leads to the presence of excess water molecules and, therefore, a condition of constant 100 % relative humidity. Liquid acetone was then added to this state by the syringe to create the desired organic solvent atmosphere. After a predefined exposure time interval, the solvent was removed by purging the chamber with a high humidity air flow created by bubbling dried nitrogen through a bottle of DI water with a flow rate of 1.8 L/min (Figure 2b). To ensure that the sample response was not influenced by the two different ways of creating a humidity environment (static and dynamic flow), control measurements have been performed with a commercial humidity sensor (Hytelog-RS232, B + B sensors, Germany). The results depicted in Figure 2c clearly indicate the stability of the created environment, regardless of the method used. Further details about the experimental setup, the calculation of injected liquid organic solvent volume and corresponding concentrations are outlined in detail in our previous work.[S4,S5]

Two testing procedures for different sample types were employed:

(i) For pure MXene samples: The sample was placed in the chamber followed by purging with a dried nitrogen flow. Subsequently, the gas flow was turned off, absolute acetone injected, and the chamber sealed. Following exposure to the organic environment, the chamber was again purged with the dried nitrogen flow before continuing to the next gas atmosphere (acetone concentration). The duration of each condition was 10 min.

(ii) For hydrogel-containing samples: This second procedure was applied for all hydrogel-based samples to ensure sufficient hydration of the polymer component. A liquid water reservoir was consistently present in the chamber throughout the test to generate water vapor. The sample was initially placed in the chamber, which was then rinsed with a humid gas flow produced by the bubbler. In the subsequent step, the gas flow was stopped, absolute acetone injected, and the chamber sealed. After exposure to the organic gas, the chamber was purged again with the same humid gas flow before moving on to the next acetone condition. In this study, all gas conditioning intervals, including exposure to the organic atmosphere and purging of the chamber, were set to 10 min.

In both testing cases, the IDE resistance was measured by a digital multimeter (Fluke 45 Dual Display Multimeter, USA) and the output results recorded with a self-programmed script (one data point every 2 s). The gas flow rate was measured and controlled by a mass flow controller (Bronkhorst EL-flow with a nominal flow rate of 5 l/min).

WILEY-VCH

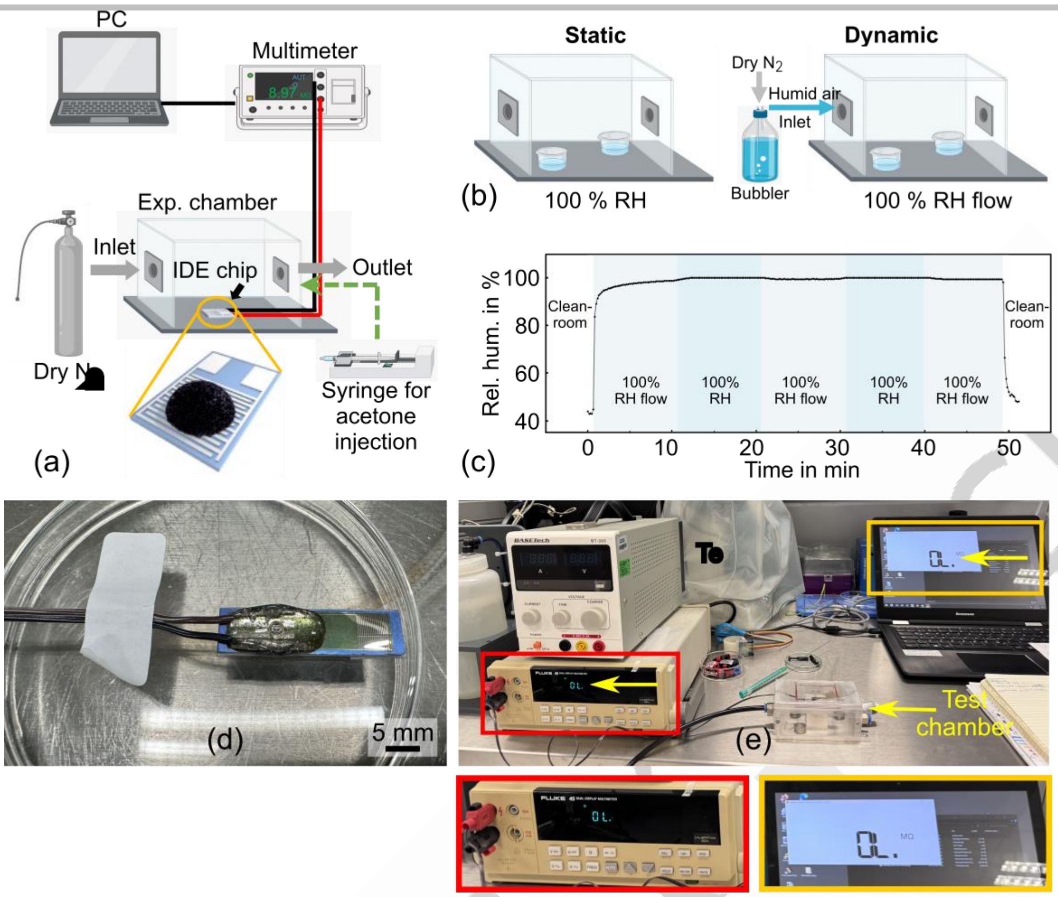

Figure S2. Setup for IDE characterization: a) gas sensing test, b) setup modification for creation of a static saturated water vapor atmosphere (left) and dynamic humid air flow (right), c) baseline measurement of the relative humidity in the chamber created by either static conditions or dynamic humidity flow (see subfigure b), d) bare IDE without any sample and e) its test results in different environmental conditions (same humidity and acetone protocol as in the chemiresistive sample analysis). Due to the absence of a conductive material, the output signal showed "OL" for all conditions.

a

$$\frac{c}{\mathrm{ppm}} = \frac{\frac{24.22}{\frac{\mathrm{L}}{\mathrm{mol}}} \cdot \frac{T}{\mathrm{K}} \cdot \frac{\rho_f}{\frac{\mathrm{g}}{\mathrm{mL}}} \cdot \frac{V_f}{\mathrm{mL}}}{\frac{273}{\mathrm{K}} \cdot \frac{M}{\frac{\mathrm{g}}{\mathrm{mol}}} \cdot \frac{V}{\mathrm{L}}} \cdot 10^3$$

24.22 L mol^{-1}……ideal gas volume at 22 °C (295 K) and one standard atmosphere pressure (101.325 kPa; 760 mm Hg)

273 K……equals 0 °C in Kelvin scale

c……gaseous analyte concentration (ppm)

T……working temperature in K (295 K in cleanroom)

ρ_f……density of the liquid organic solvent (g mL^{-1})

V_f……volume of liquid solvent (mL)

M……the molecular weight of the analyte (g mol^{-1})

V…….the volume of the testing chamber in liter

b

Acetone concentration	20 ppm	60 ppm	100 ppm
Injected liquid acetone	0.02 mL	0.06 mL	0.10 mL

Figure S3. Determination of the amount of liquid acetone for reaching predefined concentrations: a) Calculation formula and corresponding parameters, b) volume of the injected liquid acetone. For details refer to reference [S4].

3. Supporting Figures

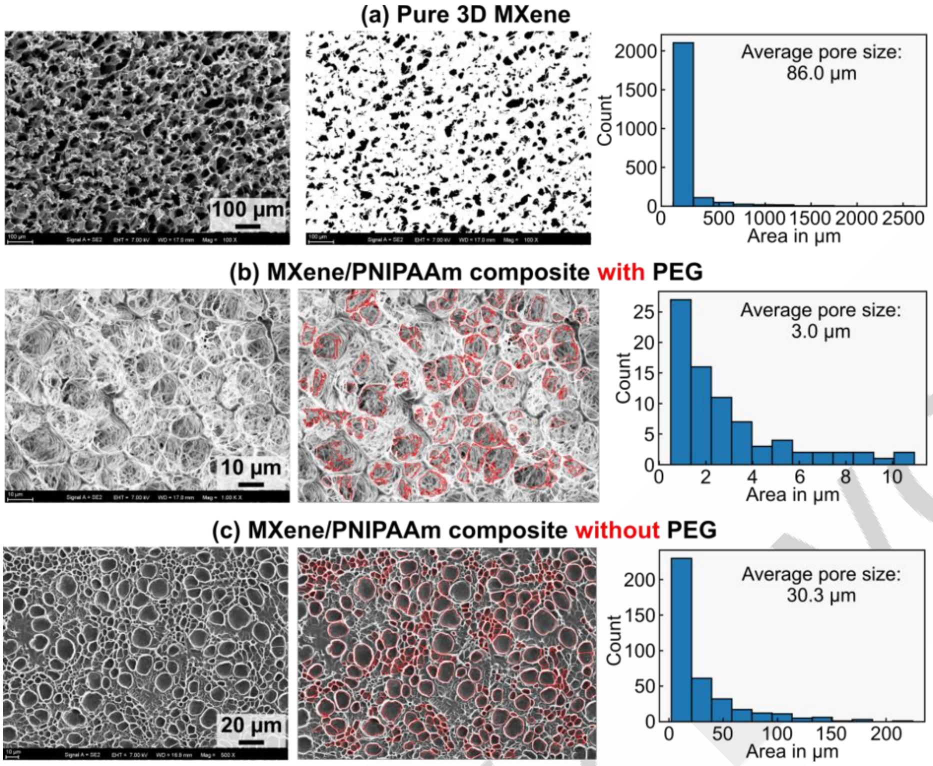

Figure S4. Exemplary SEM images and *ImageJ* evaluation of pore size and distribution for (a) pure 3D Mxene, (b) MXene/PNIPAAm composite with the porogen PEG, and (c) composite without PEG. The latter is included for completeness but has not been studied further in the presented work due to the absence of an interconnected porous structure through the bulk of the material. All observed pores are surface voids that do not penetrate into the body of the material.

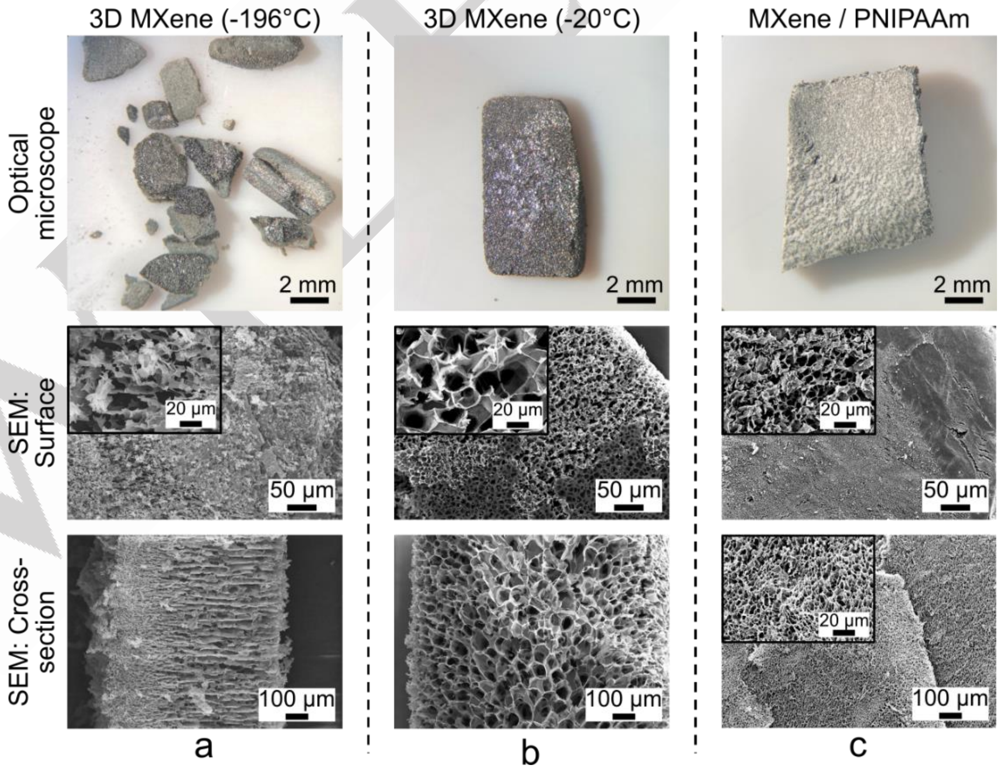

Figure S5. Optical microscope and SEM images (surface and cross section) of freeze-dried samples: a) pure MXene fabricated by ice-templating at -196 °C. b) Pure MXene fabricated by ice-templating at -20 °C. c) MXene/PNIPAAm composite fabricated at room temperature. All samples were freeze-dried using liquid nitrogen.

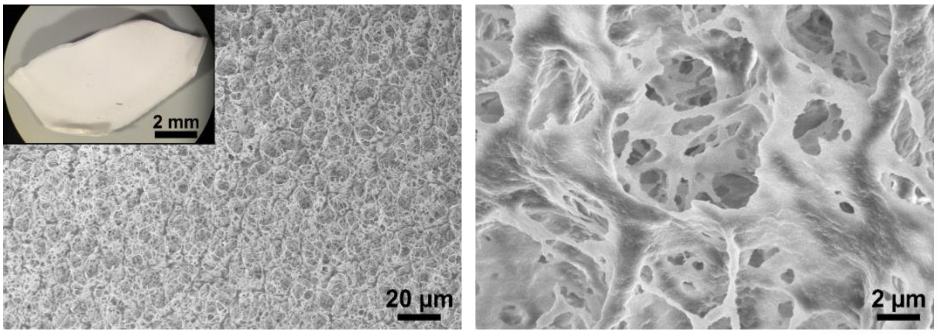

Figure S6. Optical microscope and SEM images (left: surface, right: cross section) of freeze-dried (by liquid nitrogen) pure PNIPAAm hydrogel with PEG as porogen that was washed out after polymerization.[S5]

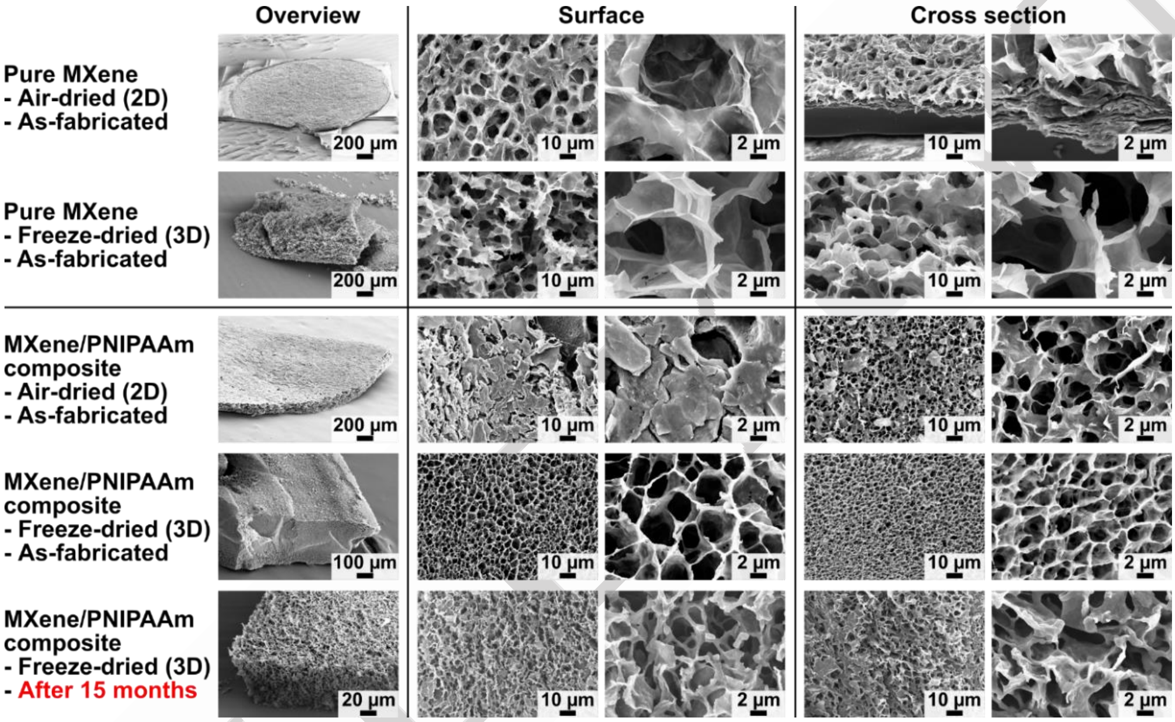

Figure S7. Detailed SEM images of all on-chip sample types (air-dried, freeze-dried, pure MXene, MXene/PNIPAAm composite). For the composite, as-fabricated and appearance after 15 months of repeated testing and storage are shown. An overview (left) and a larger magnification (right) are depicted in the *surface* and *cross section* columns.

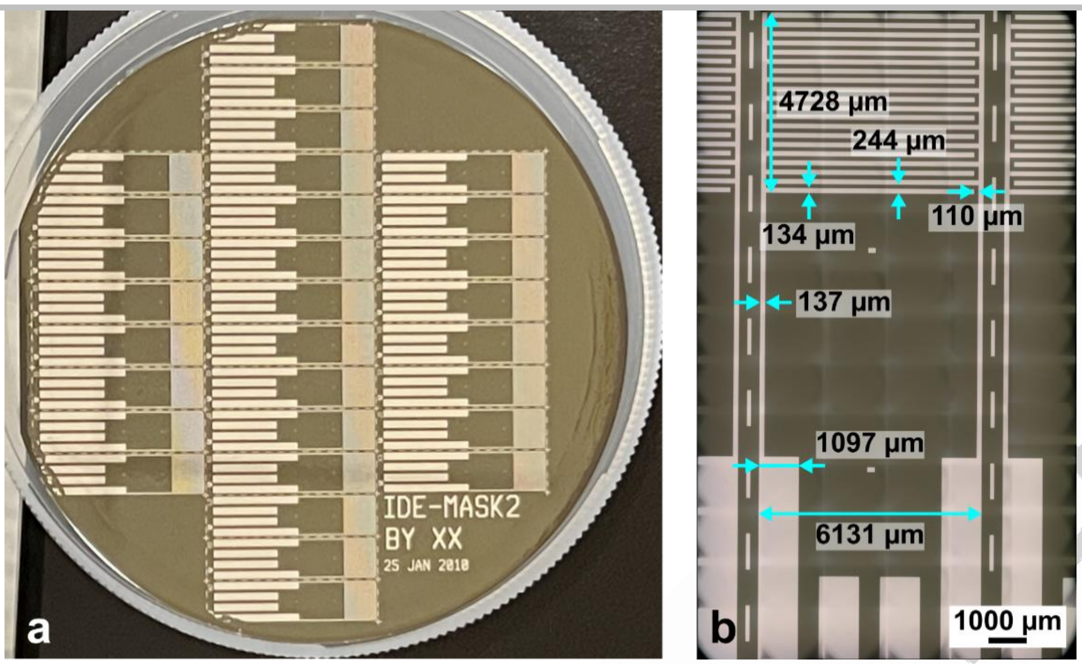

Figure S8. a) Platinum IDEs on polyimide on a 4” silicon carrier wafer (photographic image). b) Dimensions of one IDE electrode. IDEs were fabricated by Benozir Ahmed from the Department of Electrical and Computer Engineering at the University of Utah, USA.

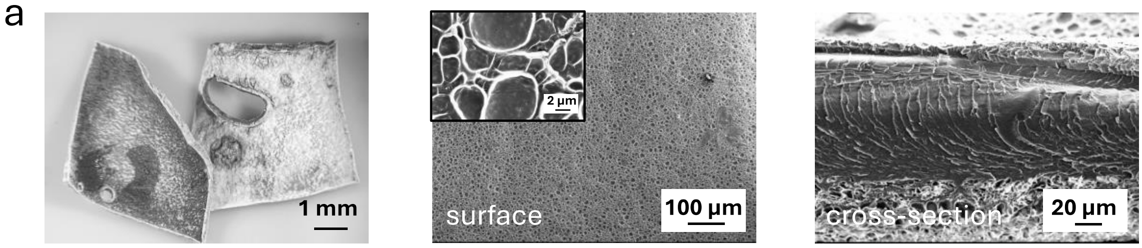

NIPAAm	BIS	APS	TEMED	MXene suspension	DIW
0.25 g	0.33 g	0.0167 g	7.5 µL	1 mL (10 mg/mL)	2 mL

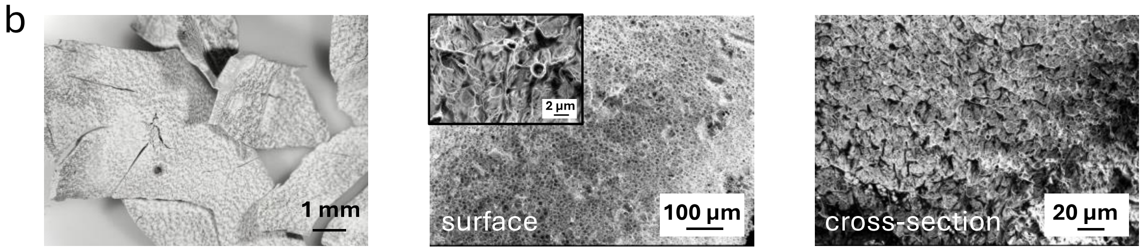

NIPAAm	BIS	APS	TEMED	PEG	MXene suspension	DIW
0.25 g	0.33 g	0.0167 g	7.5 µL	0.5 g	1 mL (10 mg/mL)	2 mL

Figure S9. Synthesis recipes and optical as well as SEM images (surface, cross-section) of MXene/PNIPAAm composites. a) Plain MXene/PNIPAAm fabricated by mixing of NIPAAm precursor and MXene suspension. b) PEG-modified MXene/PNIPAAm with PEG as porogen that is washed out after polymerization.

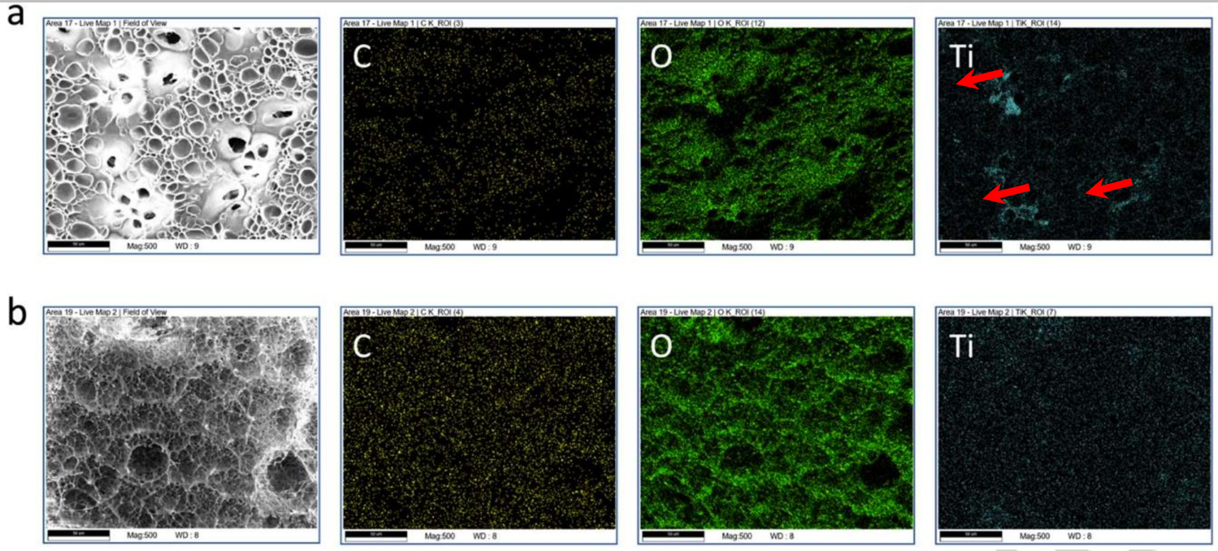

Figure S10. Energy-dispersive X-ray (EDX) spectroscopy mapping results of MXene/PNIPAAm composites: a) Unmodified (without porogen) and b) PEG-modified. In both cases, the samples were fabricated from a MXene suspension with a concentration of 10 mg/mL. The red arrows in the Ti mapping in subfigure (a) indicate titanium aggregation. This effect only occurs without the use of a porogen.

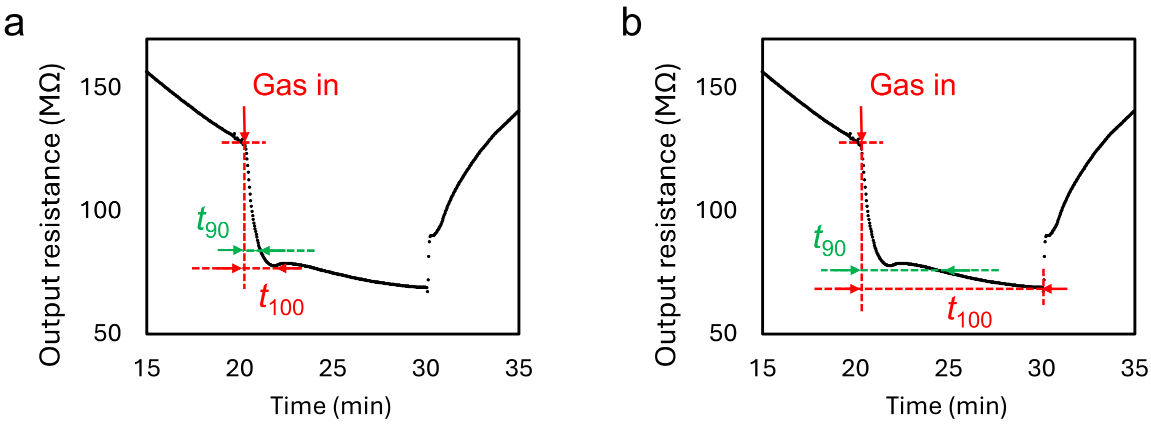

Figure S11. Determination methods of the response time t_{90}. a) Calculation based on step height of the initial sharp drop. b) Calculation based on the overall resistance decrease (90 % of steady-state value). The first method has been used for the determination of the response times listed in table S1.

4. Supporting Tables

Table S1. Calculated response time t_{90} (min) and magnitude of initial IDE resistance drop (in MΩ) according to Figure 3 (main text) and Figure S11.

Acetone concentration	100 ppm (1st)	100 ppm (2nd)	100 ppm (3rd)	60 ppm	20 ppm
Response time (acc. to S11a)	1.18	1.13	1.3	0.86	0.67
Response time (acc. to S11b)	5.84	5.5	5.71	6.58	7.24
Magnitude of initial drop (S11a)	50.3	39.6	41.1	31.8	20.7
Magnitude of initial drop (S11b)	60.8	48.1	50.7	46.5	40.6

Table S2. Timeline for repeated tests of the porous MXene/PNIPAAm composite on IDE in Figure 5 (main text) spanning 3 months.

Test date	Test sequence	Gaseous conditions
2024/01/08	1st	100/100/100/60/20 ppm (conditioning)
2024/02/23	2nd	20/60/100/60/20 ppm
2024/03/02	3rd	20/60/100/60/20 ppm
2024/03/09	4th	20/60/100/60/20 ppm
2024/04/06	5th	20/60/100/60/20 ppm

Table S3. All test data of the MXene/PNIPAAm on-chip, freeze-dried composite sample (Figure 5 in main text) for the three-month repeated testing (timeline table S2). The resistance changes ΔR (in MΩ) have been calculated based on the initial drop after the introduction of acetone. The first test is excluded as it followed a different acetone concentration cycling for conditioning.

Test / Acetone conc.	20 ppm	60 ppm	100 ppm	60 ppm	20 ppm
2nd test	2.9	5.2	8.1	7.7	4.2
3rd test	6.5	10.6	13.3	9.6	5.6
4th test	8.9	11.4	17.0	11.1	5.4
5th test	1.9	3.1	5.6	3.2	2.2

5. References

[S1] H. Chen, H. Ma, P. Zhang, Y. Wen, L. Qu, C. Li, *ACS Nano* **2020**, *14*, 10471.
[S2] G. Gao, S. Yang, S. Wang, L. Li, *Scr. Mater.* **2022**, *213*, 114605.
[S3] Z. Zhang, Z. Yao, Y. Li, S. Lu, X. Wu, Z. Jiang, *Chem. Eng. J.* **2022**, *433*, 134488.
[S4] S. Wang, G. Gerlach, J. Körner, *Polymer* **2023**, *278*, 126009.
[S5] S. Wang, G. Gerlach, J. Körner, *ACS Biomacromolecules* **2024**, *25*(5), 2715.

WILEY-VCH
